# Genetic control of the mouse HDL proteome defines HDL traits, function, and heterogeneity[S]

**DOI:** 10.1194/jlr.M090555

**Published:** 2019-01-08

**Authors:** Nathalie Pamir, Calvin Pan, Deanna L. Plubell, Patrick M. Hutchins, Chongren Tang, Jake Wimberger, Angela Irwin, Thomas Q. de Aguiar Vallim, Jay W. Heinecke, Aldons J. Lusis

**Affiliations:** Department of Medicine,* Knight Cardiovascular Institute, Oregon Health and Science University, Portland, OR; Departments of Genetics† University of California at Los Angeles, Los Angeles, CA; Biological Chemistry,** University of California at Los Angeles, Los Angeles, CA; Department of Medicine§ University of Washington, Seattle, WA

**Keywords:** high density lipoprotein, single nucleotide polymorphism, sterol efflux

## Abstract

HDLs are nanoparticles with more than 80 associated proteins, phospholipids, cholesterol, and cholesteryl esters. The potential inverse relation of HDL to coronary artery disease (CAD) and the effects of HDL on myriad other inflammatory conditions warrant a better understanding of the genetic basis of the HDL proteome. We conducted a comprehensive genetic analysis of the regulation of the proteome of HDL isolated from a panel of 100 diverse inbred strains of mice (the hybrid mouse diversity panel) and examined protein composition and efflux capacity to identify novel factors that affect the HDL proteome. Genetic analysis revealed widely varied HDL protein levels across the strains. Some of this variation was explained by local *cis*-acting regulation, termed *cis*-protein quantitative trait loci (QTLs). Variations in apoA-II and apoC-3 affected the abundance of multiple HDL proteins, indicating a coordinated regulation. We identified modules of covarying proteins and defined a protein-protein interaction network that describes the protein composition of the naturally occurring subspecies of HDL in mice. Sterol efflux capacity varied up to 3-fold across the strains, and HDL proteins displayed distinct correlation patterns with macrophage and ABCA1-specific cholesterol efflux capacity and cholesterol exchange, suggesting that subspecies of HDL participate in discrete functions. The baseline and stimulated sterol efflux capacity phenotypes were associated with distinct QTLs with smaller effect size, suggesting a multigenetic regulation. Our results highlight the complexity of HDL particles by revealing the high degree of heterogeneity and intercorrelation, some of which is associated with functional variation, and support the concept that HDL-cholesterol alone is not an accurate measure of HDL’s properties, such as protection against CAD.

HDLs are composed of a heterogeneous group of lipid-protein complexes that circulate in the blood. HDL-cholesterol (HDL-C) levels exhibit strong inverse correlations with coronary artery disease (CAD) in many populations (1, 2), and their (3, 4) abilities to promote reverse cholesterol transport and suppress inflammatory responses are consistent with the concept that they protect against the disease (5, 6). However, evidence from genome-wide association studies (GWASs) has suggested that association with CAD may not be causal (7). Certain alleles that raise HDL-C levels at GWAS loci were not associated with protection against CAD in “Mendelian randomization” studies. Additionally, clinical traits with drugs that raise HDL-C did not show protection against CAD additional to the statin effect. It has been suggested that the discrepancy may be explained by the heterogeneity of HDL (8). Thus, it is possible that certain species of HDL, but not others, provide protection against CAD, and that the genetic or drug perturbations failed to impact those mediating the protection. In particular, certain population studies have found that the efficiency of HDL in mediating cholesterol efflux from cells has been associated with decreased incidence of CAD (9–11). In addition to CAD, HDL is likely to mediate a variety of immune and regulatory functions (12–14). Because these protective functions are most likely regulated by the proteins associated with HDL, understanding the regulation of the HDL subpopulation’s proteome heterogeneity is imperative.

Based on the size, density, electrophoretic mobility, and protein content of HDL particles, subspecies of HDL have been identified in a variety of species. These include small lipid-poor HDL species as well as larger HDL species that contain a large core of cholesteryl esters (3, 4). In humans, discrete classes of HDL based on size can be identified. In mice, HDL sizes are more continuous (15) and represent one monodisperse peak. The size and levels of HDL vary in both human and mouse populations (16, 17). There are clear functional differences associated with the various size classes of HDL. In particular, the small lipid-poor particles are the best acceptors of cholesterol from cells and thus should be particularly important in mediating reverse cholesterol transport; whereas larger particles, associated with proteins such as paraoxonase 1 (PON1) and APOE, are likely to be important in protecting against inflammation. HDL particles containing proteins such as serum amyloid A (SAA) species tend to lack anti-inflammatory properties (18).

HDL-C levels have a skewed normal distribution in the general population, and the median levels vary by sex and ethnicity. Linkage-based studies from the early 1980s have tried to identify the genetic factors that influence plasma HDL-C levels, but many findings have not been replicated due to the polygenic nature of this trait, with contributions from multiple small-effect gene variants. Meta-analyses and GWAS results do, however, support the association of HDL-C with variation in CETP, LIPC, LPL, ABCA1, endothelial lipase (LIPG), and LCAT (19, 20). Multiple genetic factors could be present in an individual, creating a polygenic network of HDL-C determinants (21). These determinants include monogenic effectors such as rare homozygous mutations in ABCA1, LCAT, and APOA1 causing extremely low HDL-C (22–24) and rare homozygous mutations in CETP, LIPC, and SCARB1 causing extremely elevated HDL-C. The mouse models of these variants have been supportive of the human findings (25). Polygenic determinants have been recently investigated using targeted next-generation sequencing in patients with extremely low and high HDL-C. About 30% of individuals at the extremes of HDL-C had rare large effect and common small effect variants explaining the trait (26). Whereas the genetic determinants of plasma HDL-C levels have been well studied, the genetic determinants of the HDL proteome and lipidome have never been previously investigated.

To better define the various species of HDL at the level of protein composition and to understand their genetic regulation, we used the hybrid mouse diversity panel (HMDP) with a systems biology approach (27, 28). The HMDP is a collection of 100 classical laboratory and recombinant inbred strains that have been genotyped at 135,000 SNPs (27). The HMDP provides a confined genetic space (27) that relies on naturally occurring genetic variation that perturbs protein abundance. We performed a systems genetics approach using analytical approaches that included genome-wide association, expression quantitative trait locus (eQTL) discovery, functional outcomes, and network analysis (27).

We identified the HDL proteome and HDL function for each strain. Using quantitative trait locus (QTL) analyses, we performed genetic mapping. First, associations between SNPs and HDL protein levels and function were determined. Second, the effects of SNPs on gene regulation were determined by eQTL analysis using hepatic and adipose tissue-specific gene expression. Based on the genetic variation in HDL observed, we were able to identify numerous genetic factors mediating HDL composition and provide an approximation of the nature of the interactions between the proteins. Finally, we used protein-protein interaction cluster analyses to build an HDL model that describes the group of proteins that constitute the core proteins and the ones that are peripheral. We identified moderate QTLs associated with the sterol efflux capacity of HDL. Our results reveal a great deal of heterogeneity and intercorrelation, some of which is associated with functional variation, supporting the concept that HDL-C alone is not an accurate measure of the protective properties of HDL in terms of CAD.

## METHODS

### Mice

All studies were approved by the Animal Care and Use Committee of the University of California, Los Angeles. Mice were housed (1–3 per cage) in a pathogen-free barrier facility (22°C) with a 12 h light/dark cycle with free access to food and water. All the strains were a fed low-fat diet (Wayne Rodent BLOX 8604; Harlan Teklad Laboratory). Mice (60–80 days old) were fasted for 16 h at 7:00 PM and euthanized at 9:00 AM the following morning. Mice were bled from the retro-orbital sinus into tubes containing EDTA (final concentration 1 mM) after isoflurane inhalation. Plasma was collected and stored at −80°C until analysis. The 93 strains are represented by N = 1–5 with a distribution of ∼4, 9, 75, 9, and 1% for N = 1, 2, 3, 4, and 5, respectively (details are presented in supplemental Table S1)

### Plasma HDL-C measurements

Plasma cholesterol levels (Invitrogen) were determined biochemically following the manufacturer’s guidelines.

### Cholesterol efflux assays

Macrophage cholesterol efflux capacity was assessed with J774 macrophages labeled with [^3^H]cholesterol and stimulated with a cAMP analog, as described by de la Llera-Moya et al. (29). Efflux via the ABCA1 pathways was measured with BHK cells expressing mifepristone-inducible human ABCA1 that were radiolabeled with [^3^H]cholesterol (30). Efflux of [^3^H]cholesterol was measured after a 4 h incubation in medium with APOB-depleted serum HDL (2.8% v/v). ABCA1-specific cholesterol efflux capacity was calculated as the percentage of total [^3^H]cholesterol (medium plus cell) released into the medium of BHK cells stimulated with mifepristone after the value obtained with cells stimulated with medium alone was subtracted.

### HDL isolation

Serum HDL was prepared by adding calcium (2 mM final concentration) to plasma and using polyethylene glycol (8 kDa, Sigma) to precipitate lipoproteins containing APOB (VLDL, IDL, LDL). After centrifugation at 10,000 *g* for 30 min at 4°C, serum HDL was harvested from the supernatant. HDL was isolated from serum or EDTA-anticoagulated plasma using sequential ultracentrifugation (d = 1.063–1.21 mg/ml) (31). HDL was stored on ice in the dark and used within 1 week of preparation. For each isolation batch, control samples from the same pooled mouse plasma were included and further processed by tryptic digest and MS to control for experimental variability. The spectra for each control were monitored for peak intensities, resolution, retention times, and identified proteins.

### LC-ESI-MS/MS analysis

HDL (10 μg protein) isolated by ultracentrifugation and 0.5 ug of yeast carboxypeptidase were solubilized with 0.1% RapiGest (Waters) in 200 mM ammonium bicarbonate, reduced with dithiothreitol, alkylated with iodoacetamide, and digested with trypsin (1:20, w/w HDL protein; Promega) for 3 h at 37°C. After a second aliquot of trypsin (1:20, w/w HDL protein) was added, samples were incubated overnight at 37°C. After RapiGest was removed by acid hydrolysis, samples were dried and stored at −20°C until analysis. Prior to analysis, samples were reconstituted in 5% acetonitrile and 0.1% formic acid (17).

Tryptic digests of mouse HDL (1 μg protein) were injected onto a C18 trap column (Paradigm Platinum Peptide Nanotrap, 0.15 × 50 mm; Michrom Bioresources Inc., Auburn, CA), desalted (50 μl/min) for 5 min with 1% acetonitrile/0.1% formic acid, eluted onto an analytical reverse-phase column (0.15 × 150 mm, Magic C18AQ, 5 μm, 200 A; Michrom Bioresources Inc.), and separated on a Paradigm M4B HPLC (Michrom Bioresources Inc.) at a flow rate of 1 μl/min over 180 min, using a linear gradient of 5–35% buffer B (90% acetonitrile, 0.1% formic acid) in buffer A (5% acetonitrile, 0.1% formic acid). ESI was performed using a CaptiveSpray source (Michrom BioResources, Inc.) at a 10 ml/min flow rate and 1.4 kV setting. HDL digests were introduced into the gas phase by ESI and positive ion mass spectra were acquired with a orbitrap mass spectrometer (Fusion, Thermo Electron Corp.) using data-dependent acquisition (one MS survey scan followed by MS/MS scans of the eight most abundant ions in the survey scan) with a *m/z* 350–1,400 scan. An exclusion window of 30 s was used after two acquisitions of the same precursor ion (17, 31). Two pooled samples were included on each 96-well tryptic digestion plate (1 batch) and injected to the mass spectrometer at 70 sample intervals (1 batch), one at the beginning and one at the end of the batch. The spectra for each control were monitored for peak intensities, resolution, retention times, and identified proteins.

### Protein identification

MS/MS spectra were matched using the Comet search engine (version 2015.01 rev. 1) against a mouse UniProt database appended with yeast carboxypeptidase Y protein sequence (52,639 total sequences). The following Comet search parameters were applied: peptide mass tolerance of ±20.0 ppm allowing for C13 isotope offsets, full tryptic digest allowing up to two missed cleavages, oxidized methionine variable modification, and carbamidomethyl cysteine static modification. The search results were subsequently processed through the Trans-Proteomic Pipeline (version 4.8.0) using the PeptideProphet and ProteinProphet tools using an adjusted probability of ≥0.90 for peptides and ≥0.95 for proteins. Each charge state of a peptide was considered a unique identification (32). We used the gene and protein names in the Entrez databases (National Center for Biotechnology Information) based on the nomenclature guidelines of the Human Gene Nomenclature Committee (https://www.genenames.org/about/guidelines/) for human (33) and Mouse Genome Informatics (http://www.informatics.jax.org/mgihome/nomen/gene.shtml) guidelines (34) to identify HDL proteins and to eliminate the redundant identifications of isoforms and protein fragments frequently found in databases used in proteomic analysis (35). This approach also permits cross-referencing of proteins from different species.

### Protein quantification

Proteins were quantified using peptide spectra matches (PSMs): the total number of MS/MS spectra detected for a protein (31). Proteins considered for analysis had to be detected in ≥30 analyses (10% of the total samples) with ≥2 unique peptides. Because only a few peptides are typically measured for a given protein, these peptides might not be sufficient to define all isoforms of the protein that are present in the sample therefore, when MS/MS spectra could not differentiate between protein isoforms, the isoform with the most unique peptides was used for further analysis.

PSMs for each protein, normalized to either spiked yeast carboxypeptidase or to total PSMs for peptides from each sample, were used to calculate a normalized PSM to compare the relative protein composition of mouse strains’ HDLs (31). Supplemental Table S1 provides the total calculated PSMs for each protein, the individual peptides that identified each protein, the total number of PSMs, and relative quantification as normalized to yeast carboxypeptidase Y total PSMs or total PSMs of one sample.

### HDL particle size

HDL particle size was quantified by calibrated ion mobility analysis (36). Briefly, HDL isolated by ultracentrifugation from EDTA plasma is introduced into the gas-phase ions by ESI. Because electrophoretic mobility depends chiefly on size, ion mobility analysis data are expressed in terms of particle diameter (nanometers), which corresponds to the calculated diameter of a singly charged spherical particle with the same electrophoretic mobility (36).

### Association analyses

GWAS for protein levels and gene expression was performed using correction for population structure as described (37, 38). Loci were defined as *cis* if the peak SNP mapped within 1 Mb of gene position and *trans* if it mapped outside (*cis* significance threshold, *P* < 1.4 e-3; *trans* threshold, *P* < 6.13e-6).

### Heritability

Broad sense heritability scores were calculated for each protein using R package (sommer), using the formula H2 = genetic variance/(genetic variance + residual variance).

### Statistical analyses

Data are represented as mean ± SEM. Linear correlation among the HDL metrics of the 93 strains were assessed with Pearson correlations and the association of the proteins were assessed by Spearman correlations; both were followed with Bonferroni-Holm post hoc correction for multiple comparisons. Data were analyzed with Prism (Graph Pad Prism v. 7) and R (Cran R project R program v. 3.5.1) software.

### Data and software availability

The MS/MS datasets produced in this study are available in the PRIDE consortium (ProteomeXchange accession number PXD009473) and in the UCLA-based public database established to harbor HMDP-related data (https://systems.genetics.ucla.edu/data).

## RESULTS

### Quantitation of HDL-associated protein levels in a panel of 100 inbred strains of mice

HDL isolated from 93 strains (N = 3, for 75% of the strains) of the HMDP was subjected to LC-MS/MS (17). This list of robustly identified proteins, ranked by average abundance, is presented in **Table 1**. A full list of the proteins with PSMs per biological replicate is presented in supplemental Table S1.

**TABLE 1.** List of proteins detected in mouse HDL across the HMDP

Gene Symbol	Gene Name
Abpa7	Secretoglobin, family 1B, member 7
Ahsg	α-2-HS-glycoprotein
Alb	Albumin
Antxr1	Anthrax toxin receptor 1
Antxr2	Anthrax toxin receptor 2
Apoa1	Apolipoprotein A-I
Apoa2	Apolipoprotein A-II
Apoa4	Apolipoprotein A-IV
Apoa5	Apolipoprotein A-V
Apob	Apolipoprotein B
Apoc1	Apolipoprotein C-I
Apoc2	Apolipoprotein C-II
Apoc3	Apolipoprotein C-III
Apoc4	Apolipoprotein C-IV
Apod	Apolipoprotein D
Apoe	Apolipoprotein E
Apoh	Apolipoprotein H
Apom	Apolipoprotein M
Apon	Apolipoprotein N
Arsg	Arylsulfatase G
Bpifa2	BPI fold-containing family A member 2
C3	Complement component 3
C4b	Complement component 4B (Chido blood group)
C4bpa	C4b-binding protein
Camp	Cathelicidin antimicrobial peptide
Cd97	CD97 antigen
Clec14a	C-type lectin domain family 14, member a
Clu	Clusterin
Cst6	Cystatin E/M
Ctsd	Cathepsin D
Dmkn	Dermokine
Egfr	Epidermal growth factor receptor
F10	Coagulation factor X
Fga	Fibrinogen α chain
Fgb	Fibrinogen β chain
Fgg	Fibrinogen γ chain
Gc	Group specific component
Gm5938	Predicted gene 5938
Gm94	Predicted gene 94
Gpld1	Glycosylphosphatidylinositol specific phospholipase D1
Grn	Granulin
H2-Q4	Histocompatibility 2, Q region locus 4
H2-Q10	Histocompatibility 2, Q region locus 10
Hba	Hemoglobin α chain complex
Hbb-b1	Hemoglobin, β adult major chain
Hbb-b2	Hemoglobin, β adult minor chain
Hbbt1	β-Globin
Icam1	Intercellular adhesion molecule 1
Ifi27l2b	Interferon, α-inducible protein 27 like 2B
Igfals	Insulin-like growth factor binding protein, acid labile subunit
Ighm	Immunoglobulin heavy constant mu
Ihh	Indian hedgehog
Itgb1	Integrin β 1 (fibronectin receptor β)
Lcat	Lecithin cholesterol acyltransferase
Mug1	Murinoglobulin 1
Napsa	Napsin A aspartic peptidase
Obp1a	Odorant binding protein Ia
Pcyox1	Prenylcysteine oxidase 1
Pf4	Platelet factor 4
Plg	Plasminogen
Pltp	Phospholipid transfer protein
Pon1	Paraoxonase 1
Pon3	Paraoxonase 3
Ppic	Peptidylprolyl isomerase C
Psap	Prosaposin
Rab1a	Ras-related protein Rab-1a
Rbp4	Retinol binding protein 4, plasma
Saa1	Serum amyloid A 1
Saa2	Serum amyloid A 2
Saa4	Serum amyloid A 4
Scgb1b2	Secretoglobin, family 1B, member 2
Sell	Selectin, lymphocyte
Serpina1a	Serine (or cysteine) peptidase inhibitor, clade A, member 1A
Serpina1e	Serine (or cysteine) peptidase inhibitor, clade A, member 1E
Serpina3k	Serine (or cysteine) peptidase inhibitor, clade A, member 3K
Serpinc1	Serine (or cysteine) peptidase inhibitor, clade C (antithrombin), member 1
Tf	Serotransferrin
Tfpi	Tissue factor pathway inhibitor
Tfrc	Transferrin receptor
Ttr	Transthyretin
Vcam1	Vascular cell adhesion molecule 1
Vtn	Vitronectin

**Fig. 1.**
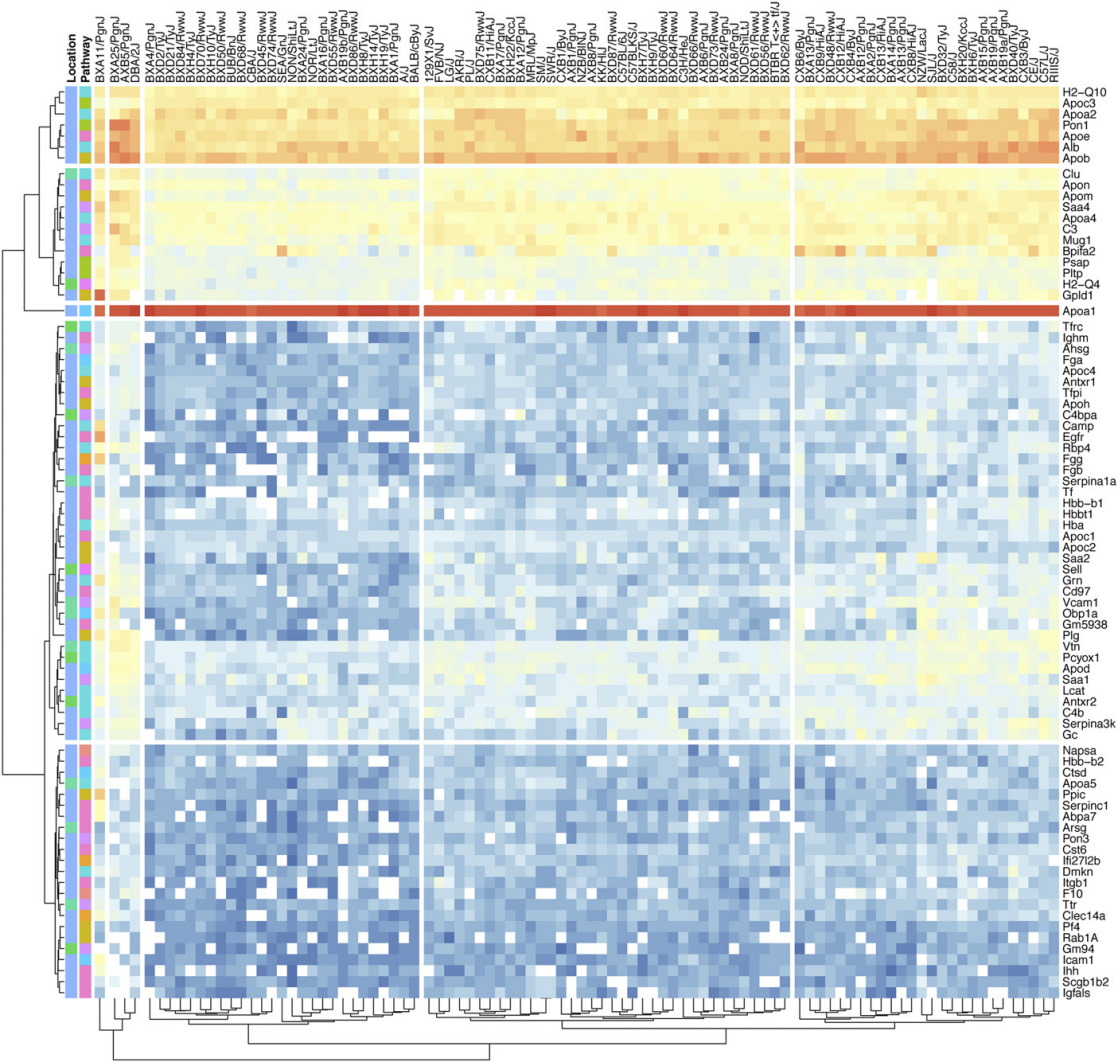
The heatmap visualization of the HDL protein abundances across 93 strains. The proteins, their biological functions, and their cellular locations are represented. Logarithmic (base 10) transformation of the yeast normalized data has been performed to accommodate the abundance distribution from high (red) to low (blue). White squares represent values that are not available. Both the proteins and the strains were clustered using Euclidean distances.

**Fig. 2.**
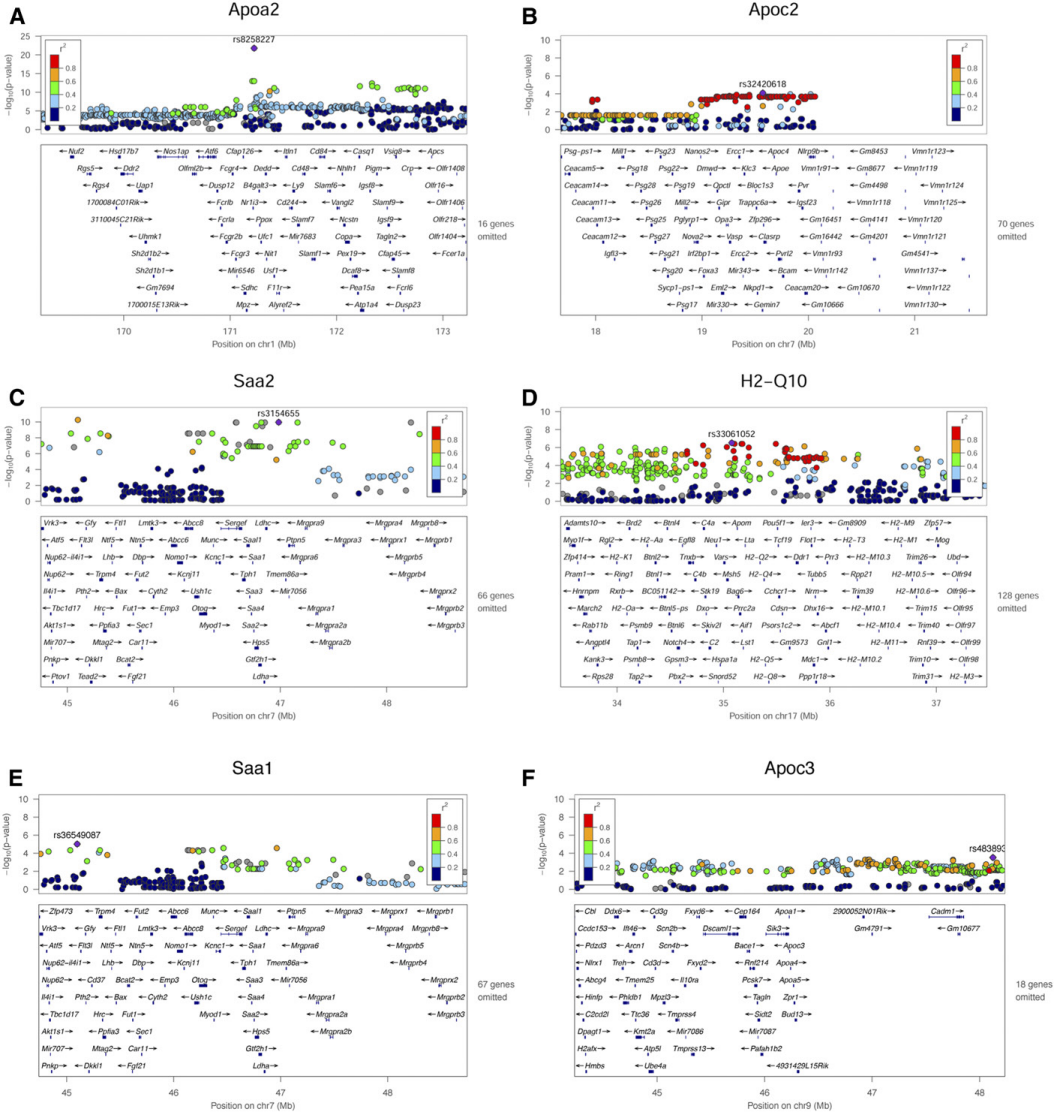
Representative QTLs for HDL proteins. Loci associated with protein levels of APOA2 (A), APOC2 (B), SAA2 (C), H2-Q10 (D), SAA1 (E), and APOC3 (F).

We used two common ways to normalize the PSMs: *1*) normalized each PSM to spiked yeast carboxypeptidase Y protein (39, 40); and *2*) normalized every PSM to the observed total PSMs (31). We present findings from both analyses: The main text data and figures are from yeast normalized data analysis, whereas the total normalized analysis is presented in the supplements. Proteins that were identified with at least two unique peptides were subjected to further analysis. Of these proteins, 34 were shared across all strains (**Fig. 1**, supplemental Fig. S1). The proteome was analyzed for proteins represented in strains by quintiles. The bottom quintile proteins, identified in less than 20% of the 93 strains, were discarded. The remaining 155 proteins identified were used to perform the QTL analyses (**Fig. 2**, supplemental Fig. S2). To identify how the proteins correlated with each other and with the functional metrics of HDL, we used proteins that were represented in 80% of the strains (81 proteins total).

The heatmap representation of the log transformed relative abundance of proteins across 93 strains, the biological processes in which they participate, and their cellular locations are visualized to provide information about the relative abundance and their spatial (Euclidean) clustering (Fig. 1, supplemental Fig. S1; the variance for each protein across the strains is presented in supplemental Fig. S8). We have curated outputs from publicly available enrichment databases, such as DAVID, PANTHER, KEGG, and Gene Ontology, to classify the proteins according to the biological functions in which they participate and the cellular location they are most likely to operate. As expected, most of the proteins (65/81) were extracellular. Our results are in agreement with previous reports (31) and show that HDL is associated with proteins that play a role in antigen processing, cell metabolism, coagulation, complement activation, immune response, lipid metabolism, metal ion binding, proteinase inhibition, and steroid binding. All of the proteins identified are replicated by previous studies, as shown by the HDL Proteome Watch website (http://homepages.uc.edu/~davidswm/HDLproteome.html) by Dr. Sean Davidson’s Laboratory at the University of Cincinnati.

Among the 155 proteins, the ones associated with highly regulated and preserved metabolic pathways, such as innate immunity and proteinase inhibition, displayed strain-specific patterns. For example, H-2 class I histocompatibility antigen (H2-Q10) was present in relatively high abundance in all the strains studied, while the histocompatibility 2 Q region locus 1 (H2-Q1) was present in 48/93 strains and H-2 class I histocompatibility antigen D-D α chain (H2-D1) is only present in 22/93 strains (supplemental Table S1). The distinct strain-specific patterns were also observed for protease inhibition class proteins, such as alpha-1-antitrypsin 1-2 (Serpin1b) present in 25/93 strains versus alpha-1-antitrypsin 1-4 (Serpina1d) present in 46/93 strains and serine protease inhibitor A3K (Serpina3K) present in 92/93 strains studied.

We had previously shown (in a smaller study with five inbred strains) that the HDL proteome predicts the genealogy of the strains (17), suggesting a hereditary component. The heritability of the HDL proteome was estimated by calculating the broad-sense heritability scores (H2) for each protein (supplemental Table S1). Broad-sense heritability (H2) captures the phenotypic variation due to genetic factors such as dominance and epistasis (41). The HMDP consists of 29 “classic” inbred strains and about 70 recombinant strains derived from five different sets of parental strains (BxA or AxB, BxD, CxB, and BxH) (27). Our cohort has N = 1–5 biological replicates per strain. The proteins identified are not always represented in each sample, leading to variable numbers. Despite the incrementally variable genetic canvas of the strains and the technical variability in MS analyses, 66/155 yeast-normalized proteins had an H2 score between 0.10 and 0.90, indicating up to 90% heritability due to genetic factors. The HDL proteome is composed of a core set of proteins (∼40) that are detected in every study across the diverse laboratories and sample sets, and a subset of proteins that are acutely regulated by the environment (i.e., diet and inflammation; http://homepages.uc.edu/~davidswm/HDLproteome.html). The H2 scores for such proteins are expected to be zero. Furthermore, due to our study design, the proteins that are strain specific are also expected to have an H2 of zero.

### Genetic regulation of the HDL proteome

Loci contributing to variations in protein levels [protein QTLs (pQTLs)] or hepatic transcript levels of the proteins (eQTLs) were mapped using the FAST LMN, an association algorithm with a mixed model component to correct for population structure. Association analysis was performed using about 200,000 informative SNPs (27), spaced throughout the genome (**Table 2**). Hepatic transcript levels were from a previous survey of the HMDP maintained on the same chow diet as in this study (27). Loci averaged from 500 kb to 2 Mb in size and, in most cases, contained 1–20 genes within a linkage disequilibrium block, an improvement of more than an order of magnitude as compared with traditional linkage analysis in mice (typically a resolution of 10–20 Mb) (42). Loci mapping within 1 Mb of the gene are termed “local” QTL, suggesting that they probably act in *cis.* For example, promoter or enhancer variants would act in *cis*. Loci mapping greater than 1 Mb from the gene of interest are termed “distal”, implying that they act in *trans*, presumably mediated by a diffusible factor, such as a transcription factor (Table 2, supplemental Table S2). We applied a significance filter of *P* = 10^−3^ and 10^−6^ to identify suggestive *cis* and *trans* QTLs, respectively.

**TABLE 2.** Local hepatic eQTLs and HDL protein pQTLs

Gene Symbol	Gene Location	Local eQTLs			Local pQTLs		
		SNP ID	Location	*P*	rsID	Location	*P*
Antxr1	chr6:87133854-87335775	rs37023898	chr6:87529811	1.81E-04	—	—	—
Antxr2	chr5:97884688-98030962	rs33642547	chr5:97956955	7.35E-09	—	—	—
Apoa2	chr1:171225054-171226379	rs46114424	chr1:170988751	1.28E-04	rs8258227	chr1:171225758	1.63E-22
Apoc2	chr7:19671584-19681423	rs32193511	chr7:19542617	2.54E-04	rs32420618	chr7:19571593	7.92E-05
Apoc3	chr9:46232933-46235636	—	—	—	rs48945377	chr9:46673334	5.41E-04
Apoh	chr11:108343354-108414396	rs29467866	chr11:107673524	8.33E-16	—	—	—
Apom	chr17:35128997-35132050	rs33061052	chr17:35078160	4.57E-11	—	—	—
Apon	chr10:128254131-128255901	rs29339020	chr10:128283501	2.27E-11	—	—	—
Arsg	chr11:109473374-109573330	rs27052095	chr11:109531899	6.33E-07	—	—	—
B2m	chr2:122147686-122153083	rs27439233	chr2:121957890	1.02E-14	—	—	—
Clu	chr14:65968483-65981548	rs31035575	chr14:66419322	5.69E-04	—	—	—
Dmkn	chr7:30763756-30781066	rs8236440	chr7:30734739	5.42E-04	—	—	—
Gpld1	chr13:24943152-24990753	rs30001837	chr13:25375000	1.63E-05	—	—	—
H2-Q10	chr17:35470089-35474563	rs33079924	chr17:35760448	7.03E-54	rs33061052	chr17:35078160	2.99E-07
Itgb1	chr8:128685654-128733200	rs33614001	chr8:128711717	1.73E-04	—	—	—
Pltp	chr2:164839518-164857711	rs27317491	chr2:164014231	5.33E-06	—	—	—
Pon1	chr6:5168090-5193946	rs30319905	chr6:4188552	9.52E-06	—	—	—
Saa1	chr7:46740501-46742980	rs32054038	chr7:47248355	4.11E-04	rs3154655	chr7:46985523	1.19E-05
Saa2	chr7:46751833-46754314	rs32054038	chr7:47248355	1.53E-06	rs3154655	chr7:46985523	1.09E-10

Local eQTLs and pQTLs with *P* < 0.001 are listed along with SNP identifiers and their locations.

A total of 19 HDL proteins showed significant evidence of local regulation of hepatic transcript levels or protein levels (Table 2). With the exception of *Apoc3*, all of the significant pQTLs also exhibited significant eQTLs, indicating that genetic variation in protein levels was largely due to regulation of gene expression. In the case of *Apoc3*, while there was no significant eQTL in liver, there was a highly significant eQTL in adipose tissue (*P* = 1.8 × 10^−7^) (supplemental Table S3). *Apoa2* and *Saa2* exhibited more significant pQTLs than eQTLs (*P* = 1.324e-22, effect size = −0.413 vs. *P* = 1.276e-4, effect size = 0.0210 for Apoa2 and *P* = 2.559e-10, effect size = 0.0780 vs. *P* = 1.533e-6, effect size = 0.780 for Saa2, respectively), suggesting that the pQTLs were due to coding rather than regulatory variations. In the case of *Apoa2*, our previous studies indicated that structural variation affecting translation efficiency was largely responsible for the differences in protein levels among several common inbred strains (27). Also, common coding variants are present among the HMDP strains for both *Apoc3* and *Saa2* (www.informatics.jax.org). As shown in Table 2, many variants significantly affecting HDL protein expression did not exhibit corresponding variations in protein levels. A likely explanation in the case of HDL is that, for some proteins, only a limited amount of the protein can be incorporated into the HDL lipid-protein complex, the remainder presumably being degraded.

We have attempted to identify distal (*trans*-acting) factors affecting HDL protein levels (supplemental Table S2). In contrast to local eQTLs, where only SNPs within 1 Mb of the gene are tested for association, distal QTL analyses involve genome-wide SNP tests for association, requiring a much higher threshold for significance. A number of the likely significant distal eQTLs occur with several megabases of the gene and, thus, probably result from either long-range (>1 Mb) linkage disequilibrium or chromosome looping interactions (for example, *Apoh*, *Apom*, *B2m*, *H2-Q10*, and *Pp1c*) (supplemental Table S2). The pQTL analysis also identified some highly significant distal (*trans*-acting) interactions, most notably for *Apoa2* (*P* = 4.6 × 10^−14^). The *Apoa2* locus is about 5 Mb from the structural gene and the significant association is probably the result of linkage disequilibrium or chromosome looping.

We asked whether the local pQTLs could be used to identify causal interactions between HDL proteins. For this, we selected the genes with significant pQTLs and asked whether the peak local pQTL SNP was associated with any other HDL proteins, suggesting that the regulation of the former perturbs levels of the latter. For example, *Apoe3* (on chr. 9) protein levels were controlled by a local pQTL with peak SNP 46673334 (chr. 9) and the same SNP was significantly associated with the levels of *Podx1* (chr. 6, *P* = 4 × 10^−3^), *Fetub* (chr. 16, *P* = 5.5 × 10^−3^), *Apoc2* (chr. 7, *P* = 5.9 × 10^−3^), and a number of other proteins. Likewise, a local pQTL SNP for *Apoa2* was associated with *Apoc3* and *Itgb3* levels and an *Hq-Q10* local pQTL SNP was associated with *Apob* and *Apoe* levels (supplemental Table S4).

### Clustering of HDL metrics based on quantitation across strains

**Fig. 3.**
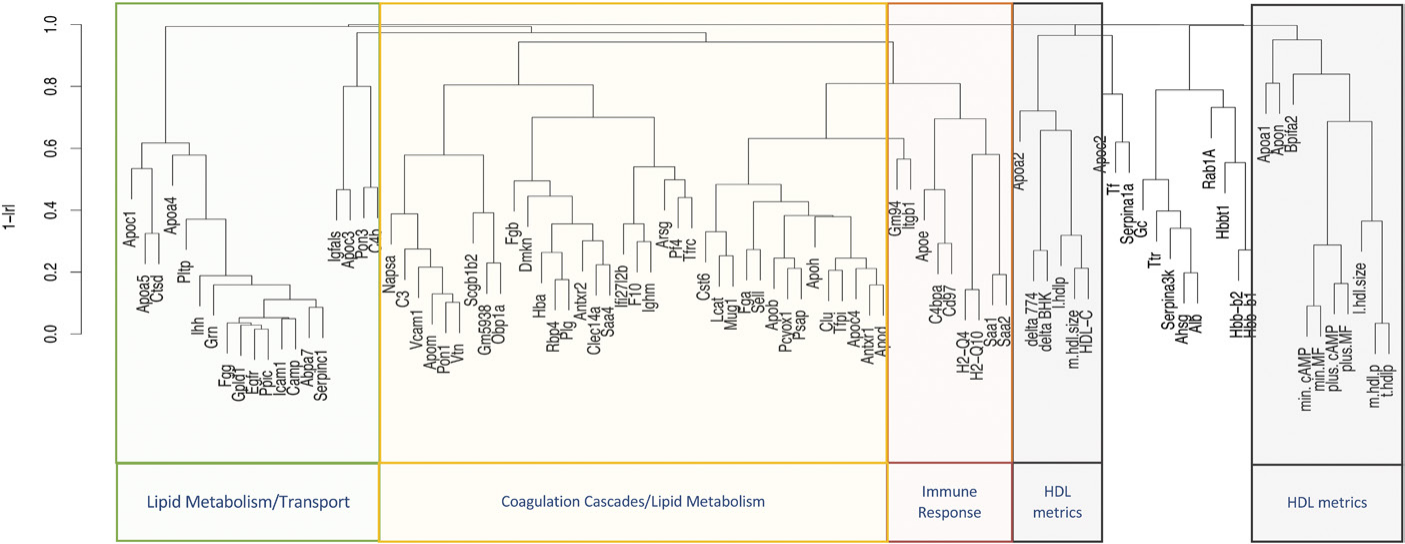
Hierarchical clustering of the HDL metrics: proteome, sterol efflux, particle concentration and size. Efflux measures for fibroblasts are min.MF (unstimulated), plus.MF (ABCA1-upregulated) delta.BHK (ABCA1-specific) and for murine macrophages are min.cAMP (unstimulated), plus.cAMP(ABCA-upregulated), and delta.J774(ABCA1-specific). m.hdl.size and l.hdl.size are medium and large HDL sizes, respectively. The correlation structure was determined using Pearson correlation. The protein functional groups were curated from DAVID, KEGG, PANTHER, and UniProt databases.

**Fig. 4.**
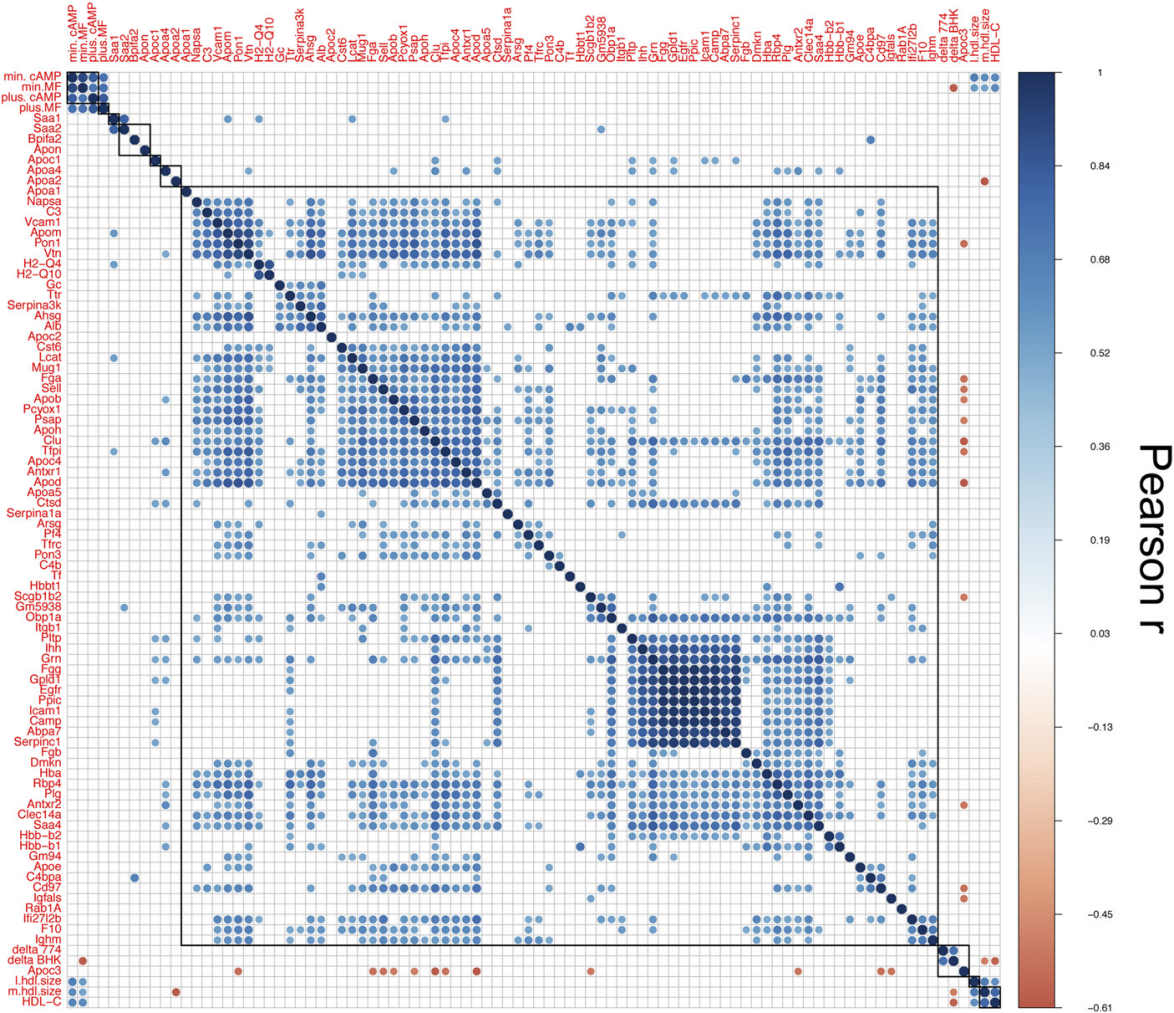
The relationship between HDL metrics is represented by a correlation matrix. A total of 8,100 correlations were observed, among which 2,216 Pearson correlations with Bonferroni-Holm correction having |r| values >0.5 (positive in blue, negative in red) that are *P* < 0.05 are presented.

To understand interactions of HDL proteins with each other and with other metrics of HDL (ABCA1-specific sterol efflux, baseline-diffusion sterol efflux, HDL particle size, and HDL-C), we correlated proteins that are present in more than 80% of the strains. We then applied hierarchical clustering to a matrix that contains all measured phenotypes. The clustering of the proteome and functional metrics revealed the expected patterns (see **Fig. 3** for yeast normalized data and supplemental Fig. S3 for total normalized data). The complex correlation matrices represented a high number of strongly correlated variables, suggesting an organized interplay among HDL proteins and between the physiological and functional metrics of HDL (see **Fig. 4** and supplemental Fig. S4 for yeast and total normalized data, respectively). Of the 8,100 total correlations, we have focused on 2,216 correlations that are |r| > 0.5 with a Bonferroni-Holm adjusted *P* < 0.05, N = 2,216. The correlation and *P* values are presented in supplemental Table S2.

All sterol efflux metrics displayed up to 4-fold differences across strains (supplemental Fig. S6). The nonproteome phenotypes clustered together; for example, unstimulated sterol efflux from two different cell types, min.cAMP and min.MF, and stimulated sterol efflux (*r* = 0.909, *P* = 10^−15^), plus.cAMP and plus.MF (*r* = 0.83, *P* = 10^−15^), correlated strongly and were part of the same cluster that is modestly associated with APOA1 (Fig. 3, supplemental Table S2). The ABCA1-mediated sterol efflux, delta BHK and delta J774, correlated strongly (*r* = 0.73, *P* = 10^−15^) and formed a cluster along with HDL-C and medium size HDL, where the latter two metrics correlated strongly (*r* = 0.78, *P* = 1.82e-12) (Fig. 3, supplemental Table S5).

HDL-C was negatively associated with ABCA1-mediated sterol efflux from both BHK and J774 cells (*r* = −0.58, *P* = 0.00036 and *r* = −0.49, *P =* 0.03, respectively) and was positively associated with the diffusional (unstimulated) efflux from both cell types (*r* = 0.62, *P* = 6.14e-06 for BHK and *r* = 0.64, *P* = 2.04e-05 for J774).

Mice have 85% of their HDL-C distributed in the 7.6–9.8 nm range (medium size) in a monodispersed peak (17); therefore, medium size HDL represents the majority of HDL-C. This latter cluster is associated with APOA2 levels (Fig. 3). Furthermore, proteins that participate in immune responses, including serum amyloids (SAA1 and SAA2) and histocompatibility complexes (H2-Q4 and H2-Q10), associated strongly and formed a distinct cluster with other immune response proteins (CD97, C4BPA, and APOE). All of the hemoglobin proteins (HBBA, HBB1, and HBBT1) formed a distinct cluster. Lipid metabolism proteins (APOC1, APOA5, APOA4, PLTP, and GPLD1) clustered together. Most interestingly, APOC3 correlated negatively with 39 proteins on HDL. Among these were proteins with roles in immune response, such as APOE, PON1, SAA1, and SAA4 (supplemental Table S5). Insulin-like growth factor binding protein, IGFALS, clustered strongly but negatively with APOC3 (*r* = −0.53, *P* = 0.002). Recent studies suggest a role for APOC3 in β cell insulin resistance (43) and according to proteome interactome by Harmonizome [a tool curated from 100 public databases (44)], APOC3 is one of the 73 proteins found to interact with IGFALS.

We observed significant correlations between HDL-C levels and GM94, ITGB1, APOC1, and APOC2 (positive) and APOA2, SERPINA1A, ALB, and TFRC (negative) regardless of PSM normalization method used (supplemental Table S5). All the New Zealand strains studied (NZB BIN/J, NZW LAC/J, and KKHI/J) were in the top quintile of HDL-C distribution and the bottom quintile of APOA2 distribution. The QTL analysis indicated the same genomic regions for regulation of APOA2 and HDL-C levels (supplemental Fig. S8). APOA2 seems to be the main genetic regulator of plasma HDL-C levels in mice.

Among the HDL proteins, APOD, with high homology to carrier proteins, such as lipocalins, and with strong innate immune response roles, such as antioxidative (45) and neuroprotective effects (46), had the most significant correlations with other HDL proteins involved in immune response. Twenty of these correlations exhibited |r| > 0.7, suggesting that it is a highly interactive apolipoprotein that acts as a carrier for other proteins on HDL.

**Fig. 5.**
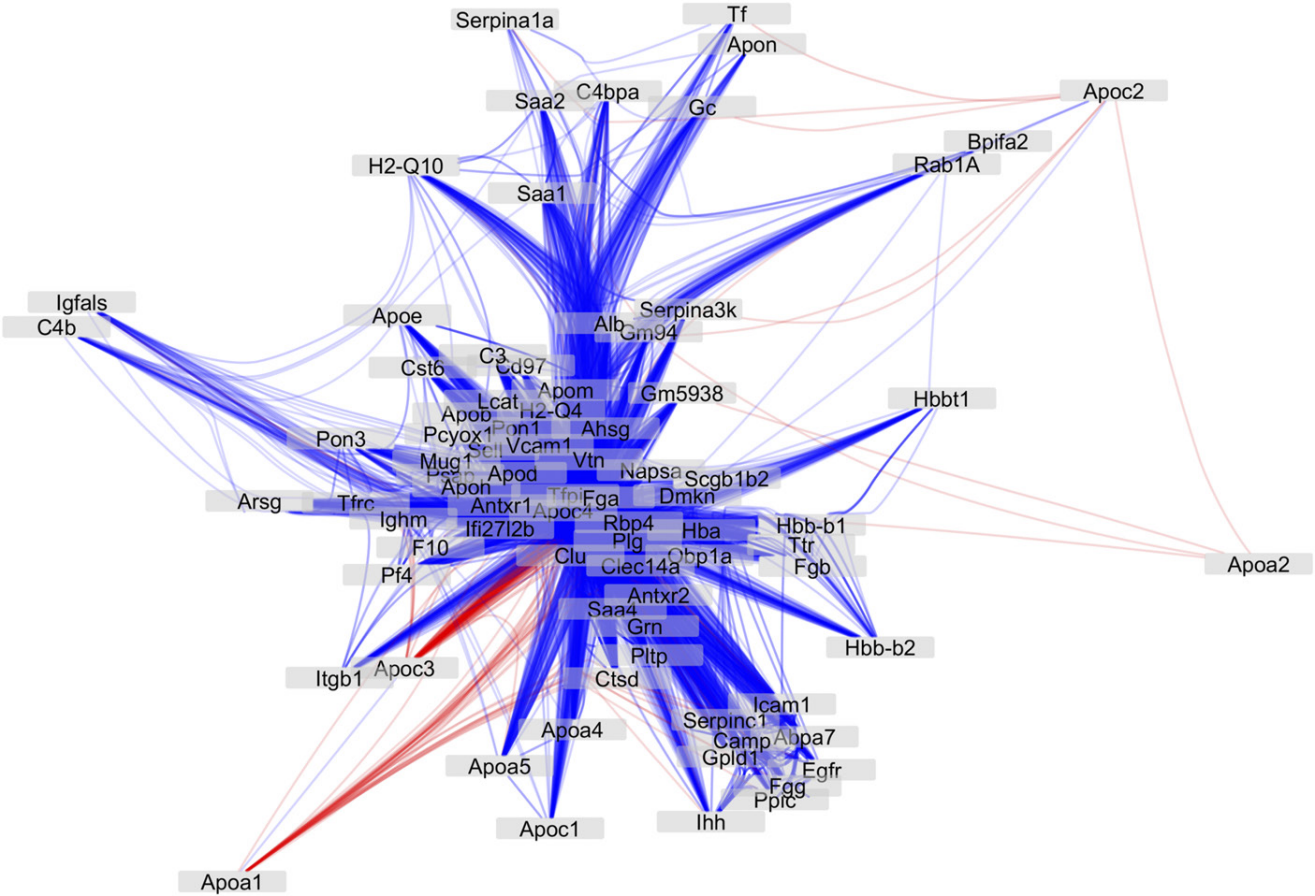
Cytoscape visualization of all the protein-protein interactions presented in Fig. 1. Self-loops were removed and edges were bundled for clarity. Node locations are assigned using an edge-weighted spring-embedded layout algorithm using the negative log of the Benjamini-Hochberg corrected *P* value, and edge transparency is directly proportional to the same value. Red, negative correlation; blue, positive correlation. Shorter distances indicate stronger correlations.

Another way to visualize the relationships among the proteins is to present them as a correlation network (**Fig. 5**, supplemental Fig. S5). The network consists of multiple layers of spatial organization, a core, an outer layer, and the periphery. The “core” proteins, including AHSG, NAPSA, plasminogen (PLG), SAA4, HBA, APOD, APOJ, APOM, APOH, FGA, and TFPI, are surrounded by common HDL-associated apolipoproteins, such as APOA4, APOA5, APOC1, APOC3, and APOC4, in addition to proteins that have been described to be associated with HDL in most proteomic studies (Davidson HDL Proteome Watch, http://homepages.uc.edu/~davidswm/HDLproteome.html), such as H2-Q4, H2-Q10, SELL, ANTRX1/2, PF4 RBP4, SERPINs, GPLD1, FGA, and FGB. More peripheral are known associated proteins such as PLTP, PON3, LCAT, APOE, SAA1, and SAA2. HDL core proteins formed a tight network, suggesting that they are coregulated. Inflammation response and complement activation proteins, such as SAA1, SAA2, H2-Q4, H2-Q10, C3, C4b, and C4BPA, were distal to the core coregulatory network, suggesting that they are mostly regulated by external factors, such as an inflammatory stimulus. The two major structural proteins, APOA1 and APOA2, along with APOC3 were on the outer layers and periphery and are negatively correlated with other proteins, suggesting that their presence requires the displacement of other proteins.

### Genetic regulation of sterol efflux

The sterol efflux capacity was measured in two different cell lines (J774 and BHK) in basal (no ABCA1) and stimulated (with ABCA1) conditions, with ABCA1-specific sterol efflux calculated by subtracting basal from stimulated. The QTL analysis for the efflux traits revealed shared global association profiles that are condition specific and in agreement between cell types. The significant QTLs that are shared by both cell types are presented in **Table 3**. The lack of strong QTLs that associate with the sterol efflux capacity of HDL suggests that either sterol efflux is under limited genetic control or its control is multigenic with small effect sizes.

**TABLE 3.** The peak QTLs for sterol efflux capacity of HDLs

SNP	CHR	BP	*P*	Beta
Baseline efflux				
rs30557586	3	55764730	4.13E-07	8.19E-01
rs31424282	3	57945327	1.74E-06	−8.38E-01
rs29379333	10	69242755	6.21E-06	8.73E-01
rs6333057	12	58206555	5.43E-07	8.76E-01
rs50224465	12	53464782	8.55E-07	1.07E+00
rs31810918	15	37462413	2.74E-08	9.80E-01
rs3718535	16	9212865	1.65E-06	7.18E-01
rs6284288	18	4398494	8.11E-06	6.97E-01
rs13483883	20	93699907	5.59E-06	8.40E-01
Stimulated efflux				
rs30557586	3	55764730	6.61E-06	6.68E-01
ABCA1-dependent efflux				
rs31551612	1	171208377	2.75E-06	−5.86E-01
rs45738488	6	67533600	4.43E-06	−6.30E-01
rs31630237	8	118112906	2.04E-07	−6.28E-01
rs26919597	11	68048005	6.78E-06	−6.30E-01
rs46923442	13	5984274	1.40E-07	−7.33E-01
rs47306105	13	6601243	2.27E-06	−7.10E-01
rs51267071	13	6240479	4.24E-06	−6.80E-01
rs46153864	13	6257029	4.24E-06	−6.80E-01
rs50517602	13	6322879	4.24E-06	−6.80E-01
rs49326176	13	6416532	4.24E-06	−6.80E-01
rs47351631	13	6520213	4.24E-06	−6.80E-01
rs46431216	13	6087220	4.48E-06	−6.82E-01
rs6373590	13	6229579	4.48E-06	−6.82E-01
rs37828224	13	8785486	9.10E-06	−6.93E-01
rs36839806	19	57424289	8.34E-07	−7.01E-01

The peak phenotypic QTL locus with *P* < 10^−6^ are listed along with SNP identifiers and their locations.

### Lipid and clinical trait interactions

The HMDP strains have been examined in separate studies (47) using chow (27), high fat/high sucrose (48, 49), or high cholesterol (48) diets. We compared our data with results from these studies, reasoning that while power to detect correlation will be diminished given that the data were collected in separate animals and at different times with incomplete overlap of strains, traits significantly impacted by genetics should still retain some correlation structure. The most significant correlations between HDL proteins and clinical traits are summarized in **Table 4** and detailed in supplemental Table S6.

**TABLE 4.** Correlations between HDL proteins and clinical traits measured within HMDP

Trait 1	Trait 2	Bicor Value	*P*
Apoc3	HOMA-IR (pre-bleed)	0.50	0.0001
Apoc1	Liver collagen	0.49	0.0011
Ttr	Liver residual	0.49	6.67E-06
Ihh	Insulin (pre-bleed)	0.47	0.0005
Plg	Lesion area	0.46	0.0002
Ighm	Lesion area	0.46	0.0003
Gm5938	Lesion area	0.45	0.0004
Apoc3	Insulin (pre-bleed)	0.44	0.0015
Ihh	HOMA-IR (pre-bleed)	0.44	0.0012
Apoc2	Kidney (left)	0.43	0.0001
Gc	Unesterified cholesterol	0.43	0.0002
Obp1a	Lesion area	0.42	0.0012
Alb	Liver residual	0.38	0.0006
Mup4	RBC	0.38	0.0007
Apoc3	Triglycerides (pre-bleed)	0.38	0.0009
Mup4	HGB	0.36	0.0014
Gc	VLDL + LDL	0.36	0.0016
Gc	Total cholesterol	0.35	0.0017
Apoc2	PLT	0.34	0.0025
Pon3	Free fluid	−0.41	0.0002
Acta2	HOMA-IR	−0.40	0.0003
Apoh	MONO	−0.37	0.0011
Ihh	Free fluid	−0.36	0.0014
Fetub	MCV	−0.35	0.0020
Scgb2b7	Adiposity	−0.35	0.0024
Acta2	Insulin	−0.34	0.0024
Apoa1	Average fat mass (liver)	−0.34	0.0025
Psap	GRAN percent	−0.34	0.0026
Apoa5	WBC time	0.34	0.0027
Acta2	Insulin	−0.34	0.0024
Psap	GRAN percent	−0.34	0.0026
Antxr1	GRAN percent	−0.34	0.0027
Gc	Triglycerides (liver)	−0.34	0.0040
Ighm	Free fatty acids (pre-bleed)	−0.34	0.0035

A total of 22 HDL proteins significantly and positively correlated with lesion area in the hypercholesterolemia study (50), among which PLG, the immunoglobin chain C region (IGHM), and platelet factor 4 (PF4) were strongest (*r* = 0.469, *P* = 0.00026; *r* = 0.461, *P* = 0.00034; *r* = 0.37, *P* = 0.0049, respectively). We have recently shown that PLG is an effective sterol acceptor through ABCA1, and this could be the mechanism by which PLG contributes to atherosclerosis (51).

Aligned with the recently identified role of APOC3 in insulin resistance, HOMA-IR, plasma insulin levels, body weight, and adiposity correlated positively with HDL-associated APOC3 levels (*r* = 0.50, *P* = 0.00017; *r* = 0.44, *P* = 0.0015; *r* = 0.23, *P* = 0.048; *r* = 0.26, *P* = 0.021, respectively).

## DISCUSSION

We report the analysis of the HDL proteome across a set of 93 inbred strains of mice exhibiting common genetic variation. The genetic variation across this mouse panel resembles that in human populations, based on the number of common SNPs (about four million). Previous studies in mice have revealed genetic variations in the levels of HDL-C, HDL apolipoproteins, and HDL composition (52). Our results are consistent with a high level of heritability of HDL proteins, including the identification of a number of novel pQTLs. One significant conclusion that has emerged is that certain proteins cluster together in response to genetic perturbations, presumably reflecting physical or regulatory interactions. These clusters then help define the heterogeneity of HDL particles, although our analyses do not address lipid heterogeneity. Another important conclusion is evidence of a relationship between protein composition and HDL function. Finally, we have identified potential links between HDL proteins and various clinical or molecular traits studied previously in the HMDP strains. We discuss each of these points in turn below.

Normalization of shotgun proteomic data is a continuous struggle in the field (53). HDL is a rather uncomplicated mixture containing only ∼100 proteins. However, its proteome is driven by the 10 most abundant proteins, with 65% being made up of APOA1 and 15% of APOA2 (54). The normalization strategy should conserve the compositional bias of the HDL. The normalization that directly adjusts scale, such as total count (TC) and upper quartile, fails to accommodate compositional bias. The normalization strategies that adjust scales using landmarks in the distribution [median (Med), differentially expressed (DESeq), and trimmed mean (TMM)] are promising approaches for the HDL proteome; however, detailed analyses need to be performed for their validation to be used on smaller libraries of stochastic count data from MS. The quartile (Q) and reads per kilobase per million mapped (RPKM) (equivalent of normalizing spectral counts to protein length) have adverse effects on intra-sample variance and on distribution bias (55). The TC and upper quartile normalizations favor the most abundant proteins and are unfriendly for mixtures with a distribution bias. That said, TC normalization is often the preferred method for shotgun HDL proteomics, as it controls for differences in instrument response, digestion efficiency, and amounts of loaded protein digest, but fails at conserving the distribution bias as it tends to accommodate the changes in the abundant proteins (31). That is partly why the HDL protein quantification by shotgun proteomics is not optimal and the correlation with immunobased assays is moderate (56). Therefore, we opted to include a second normalization approach by spiking yeast carboxypeptidase at levels ∼8-fold lower than APOA1 and to correspond to the median/mean abundance of the typical HDL proteome (39, 40). The QTLs identified using both proteomic information (stemming from two distinct normalization methods) are mostly overlapping with TC normalization, resulting in ∼30% more significant QTLs.

The relationships among inbred lines of mice were inferred from the high-density SNP map where strains cluster according to their genealogy (57). We employed the same approach: the 155 proteins that were present in at least 20% of the strains loosely predicted the relatedness of the strains according to their genealogy for inbred strains and according to the breeding scheme for recombinant strains. Almost half of these proteins (81 proteins) were present in greater than 80% of the strains and only 34 were shared by all the strains. The strain-dependent distribution of the HDL proteome across 93 strains validates our previous studies with only five strains (17). However, the comparison of the clustering patterns between microarray data or the SNPs did not reach full agreement, as genetic variation explains only a fraction of the variation, and a very small part of the genome is involved in regulating HDL (data not shown). The 93 strains are represented by N = 1–5 with a distribution of ∼4, 9, 75, 9, and 1% for N = 1, 2, 3, 4, and 5, respectively. Even though these numbers are not optimal to calculate intra- and inter-strain variation, the broad sense heritability calculations captured 65 proteins that have greater than 10% heritability; among which, APOA2 has a score of 0.62, which is consistent with its strong association with HDL-C loci, a highly heritable trait.

The high-level heritability of the proteins is demonstrated by >20,000 pQTL-associated SNPs that map to >66 loci. To understand whether a pQTL results from structural or regulatory variation, we have incorporated gene expression information. A positive finding in such analysis suggests that the genotype-dependent differential gene expression is the basis of most of the association (58). In our studies, we used adipose and liver tissue global gene expression profiles to map distinct loci in liver (5) and adipose tissue (20) that are associated with the gene expression levels for the SNPs associated with pQTLs. Adipose tissue exhibited only inflammatory gene-associated SNPs (*Saa1*, *Saa2*, *Tfrc*, and *Vtn*) that were almost exclusively *trans-*acting. Liver tissue had *cis* and *trans* eQTLs associated with multiple genes, including *Apoc4*, *Apoh*, *Fgb*, *Tfrc*, and *Saa2*. The complex regulation at the protein and gene expression level dictates the protein composition of HDL and its hereditary preservation.

Correlation networks, such as weighted gene coexpression network analysis, are a systems biology method for describing the correlation patterns among genes. The weighted correlation network analysis revealed that core HDL proteins, composed of most common apolipoproteins, are highly correlated and coregulated. The coregulated gene network is consistent with HDL’s role in innate immunity and lipid metabolism, as it reveals a tight network of coregulation among the proteins with primary roles in immunity and lipid metabolism. The histocompatibility protein isoforms, such as H2-Q4 and H2-Q10, which have been shown to be associated with mouse HDL in multiple studies (17, 59, 60), are part of the core coregulated proteins. In mice, histocompatibility proteins play a role in innate immunity by antigen presenting via major histocompatibility complex class 1. H2-Q10 is the only murine major histocompatibility complex class 1 protein found in the serum in appreciable concentrations (61). While these innate immune proteins with roles in antigen presentation are part of the core coregulation network, acute phase proteins, such SAA1 and SAA2, are not, as they are primarily regulated by an inflammatory stimulus.

While up to 50% of the HDL-C level can be heritable, less is known about heritability of its sterol efflux function or its proteome (62, 63). The sterol efflux capacity of HDL seems to be regulated by a multigenic architecture with a small effect size. The loci captured using 93 strains of mice have moderate *P* values and small effect sizes. In a human cohort of 846 individuals, Villard et al. (64) tested seven preselected SNPs with known effects in HDL metabolism, such as ABCA1, CETP, APOA1, and APOA2. The seven SNPs tested accounted together for approximately 6% of total plasma efflux capacity, supporting our findings of moderate strength QTLs.

The classic linear view of HDL genesis from discoidal lipid-poor nascent particles to spherical cholesterol- and phospholipid-rich particles packed with a combination of over 100 different proteins has been recently challenged by the finding that HDL is secreted directly from hepatocytes in four distinct sizes, with little interchange between them, and representing all of the plasma HDL subparticle pools (62). Although our analyses do not incorporate the lipidome of HDL, which can contribute to the orchestration of the composition of HDL subpopulations, a highly intercorrelated proteome reveals the complexity of HDL particle composition. We captured a remarkable 2,216 correlations among the proteins that survived multiple comparison correction, and that explains at least 25% of the variation (R2 > 0.25). The hierarchical clustering of the correlated proteins regrouped the proteins according to their biological functions, emphasizing the coordinated coregulation. The protein-protein interaction modeling identified layers of protein groups that are likely to be coregulated (Fig. 5) and that shape the HDL subparticle protein cargo. In our model, the most abundant structural proteins, APOA1, APOA2, and APOC3, correlate negatively with other protein groups, which suggests that these proteins regulate the HDL’s particle proteome. The immune response and complement cascade proteins (SAA1, SAA2, H2-Q10, C3, etc.) represented a group of proteins that are unlikely to be coregulated with weaker interactions with the core proteins, suggesting that these pathways are mostly regulated by inflammatory stimuli rather than genetic coordination.

In our studies, we applied a stringent statistical approach that led to dismissal of certain biological relationships. For example, APOC3 significantly and exclusively negatively correlated with 36 other HDL proteins (supplemental Table S2). In humans, increased circulating APOC3 levels are associated with cardiovascular disorders, inflammation, and insulin resistance (64, 65). On the other hand, humans with an APOC3 mutation benefit from a favorable lipoprotein profile, increased insulin sensitivity, lower incidence of hypertension, and protection against cardiovascular diseases (68–70). The negative correlation of APOC3 with 36 other proteins and its association with plasma insulin levels and HOMA-IR levels conforms to its newly appreciated role as a brake on the metabolic system. Efforts to identify the proteomic, lipidomic, and functional fingerprints of HDL subspecies are of critical importance and may open paths to novel pharmacological targets.

Clinical and epidemiological studies show a robust inverse association between HDL-C levels and coronary heart disease risk (2, 71). However, pharmacological interventions aimed at raising HDL-C levels in humans showed no cardiovascular benefits (72–75). Since the collapse of the HDL-C hypothesis for atherosclerosis, a new generation of HDL metrics are under investigation to be used in the clinic (76). For example, greater HDL-C efflux capacity, independent of levels of HDL-C and APOA1 (the major structural protein of HDL), is associated with a lower prevalence of atherosclerotic vascular disease (9, 10, 77). Most changes in HDL function are likely to be a reflection of changes in the HDL proteome (31, 78). Thus, identification of the protein signature responsible for the loss of sterol efflux capacity could provide biomarkers of clinical validity to assess coronary heart disease risk. The interplay between HDL sterol efflux function, particle concentration and size, and the HDL proteome is still poorly understood. HDL-C levels correlated strongly with all the efflux measures. While we captured strong associations between expected metrics such as diffusional or ABCA1-specific efflux from two different cell types, no single HDL protein explained the majority of the variation in sterol efflux, suggesting that it is a polygenic process. That said, APOA2 explained about 10% of ABCA1-dependent sterol efflux from both cell types and the *Apoa2* locus was strongly associated with ABCA1-specific sterol efflux (data not shown). In mice, APOA2 seems to impact the sterol efflux function at the protein and gene level. It is important to note that the *Apoa2* locus aligns with the HDL-C QTL.

In summary, a systems biology approach reveals the highly complex and intercorrelated nature of HDL protein composition, its heritable contributions to HDL cholesterol levels, and its association with disease. We show that HDL proteins preserve hereditary patterns that are likely to harbor ancestral/lineage information. It is likely that inheritance controls the production of HDL particles of a certain protein and lipid composition that have different functions. At present, we lack a model for the assembly of HDL protein and lipid cargo. Our results provide the ground work to support future studies aimed at characterization of the genetic architecture regulating HDL function and comprehensive composition in humans.

## Supplementary Material

Supplemental Data
