# Supplementary material for: Genetic control of the mouse HDL proteome defines HDL traits, function, and heterogeneity
Source: J Lipid Res. 2019 Jan 8;60(3):594–608. doi: 10.1194/jlr.M090555 (PMC6399512; doi:10.1194/jlr.M090555)
Supplement: Supplemental Data [file supp_60_3_594__index.html]

Genetic control of the mouse HDL proteome defines HDL traits, function, and heterogeneity — Genetic control of the mouse HDL proteome defines HDL traits, function, and heterogeneity — Supplemental Data 

# Genetic control of the mouse HDL proteome defines HDL traits, function, and heterogeneity

## Supplemental Data

- Supplemental Figures 1-8 (.pdf, 5.9 MB) - Supplemental Material
- Supplemental Table S1 (.xlsx, 3.5 MB) - The data file for all the proteomic data across the strains
- Supplemental Table S5 (.xlsx, 900 KB)
- Supplemental Table S6 (.xlsx, 63 KB)
