# Supplementary material for: Genetic control of the mouse HDL proteome defines HDL traits, function, and heterogeneity
Source: J Lipid Res. 2019 Jan 8;60(3):594–608. doi: 10.1194/jlr.M090555 (PMC6399512; doi:10.1194/jlr.M090555)
Supplement: Supplemental Data [file 10.1194_M090555_jlr.M090555-1.pdf]

Supplemental Material for “Genetic Control of HDL Proteome”.

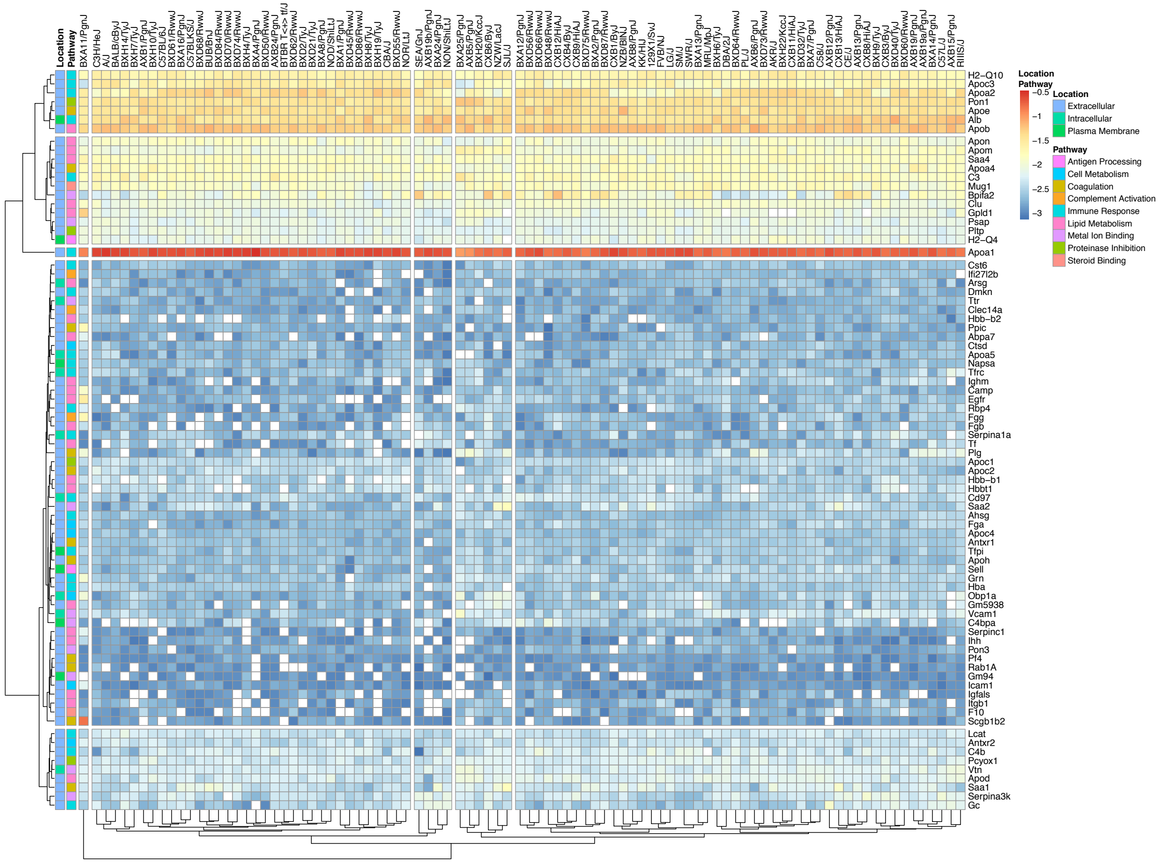

Supplemental Figure 1: The heatmap visualization of the HDL protein abundances (calculated as normalized to total PSMs) across 93 strains. The proteins, their biological functions and cellular locations are represented. Logarithmic transformation of the total PSM normalized data has been performed to accommodate the abundance distribution form high (red) to very low (dark blue). White squares represent not available values. Both the proteins and the strains were clustered using Euclidean distances.

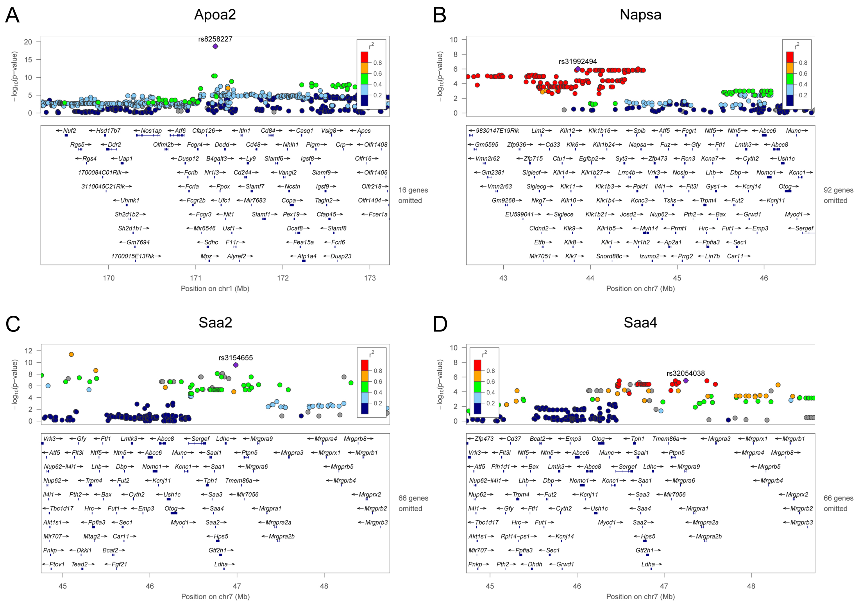

Supplementary Figure S2. A representative QTLs for HDL proteins normalized to total PSMs. Loci associated with A) APOA2, B), NAPSA, C) SAA2, D) SAA4. E) F)

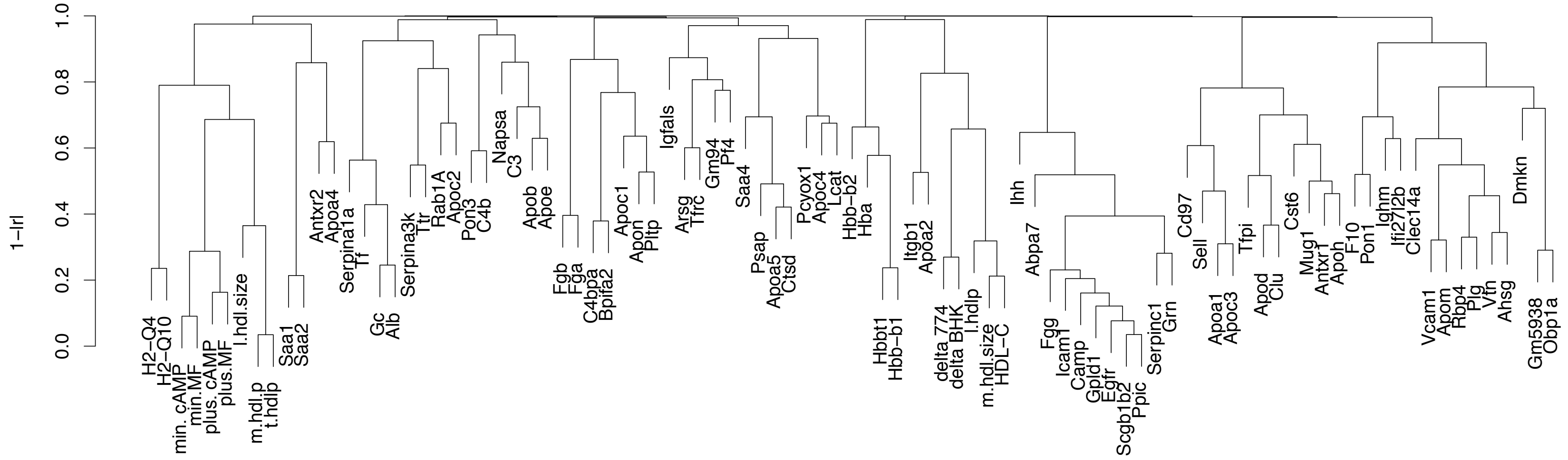

Supplementary Figure S3. Hierarchical clustering of the HDL metrics: Proteome (normalized to total PSMs), sterol efflux, particle concentration and size. min.MF, plus.MF, delta.BHK are unstimulated, ABCA1 upregulated and ABCA1 specific sterol efflux from BHK cells respectively. min.cAMP, plus.cAMP, delta.J774 are unstimulated, ABCA1 upregulated and ABCA1 specific sterol efflux from J774 cells respectively. m.hdlp and t.hdlp are medium size and total HDL particle concentration. m.hdl.size and l.hdl.size are medium and large HDL sizes respectively. The correlation structure was determined using pearson correlation. The protein functional groups were curated from DAVID, KEGG, Panther, and Uniprot databases.

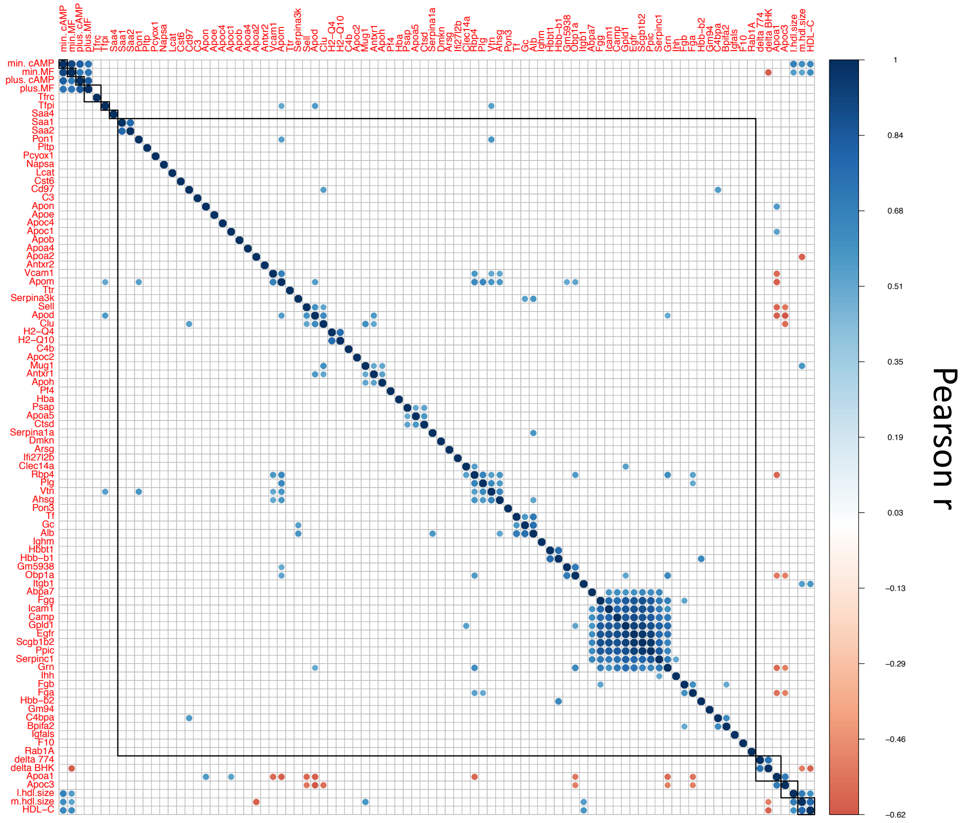

Supplementary Figure S4. The relationship between HDL metrics is represented by a correlation matrix. Total of 8,100 correlations were observed. Among which 380 Pearson correlation with Bonferroni-Holm correction having |r| values >0.5 (positive in bleu, negative in red) that are *P*<0.05 are presented.

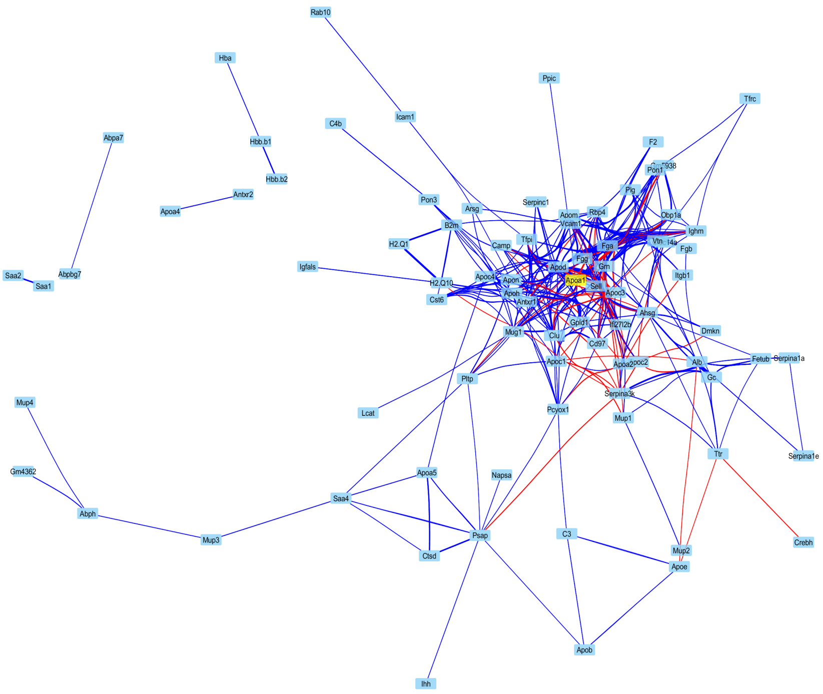

Supplemental Figure S5. Cytoscape visualization of all the protein-protein interactions presented in Supplemental Figure 2. Self-loops were removed and edges were bundled for clarity. Node locations are assigned using an edge-weighted spring-embedded layout algorithm using the negative log of the Benjamini-Hochberg corrected p-value, and edge transparency is directly proportional to the same value. Red: negative correlation, Blue: positive correlation. Shorter distances indicate stronger correlations.

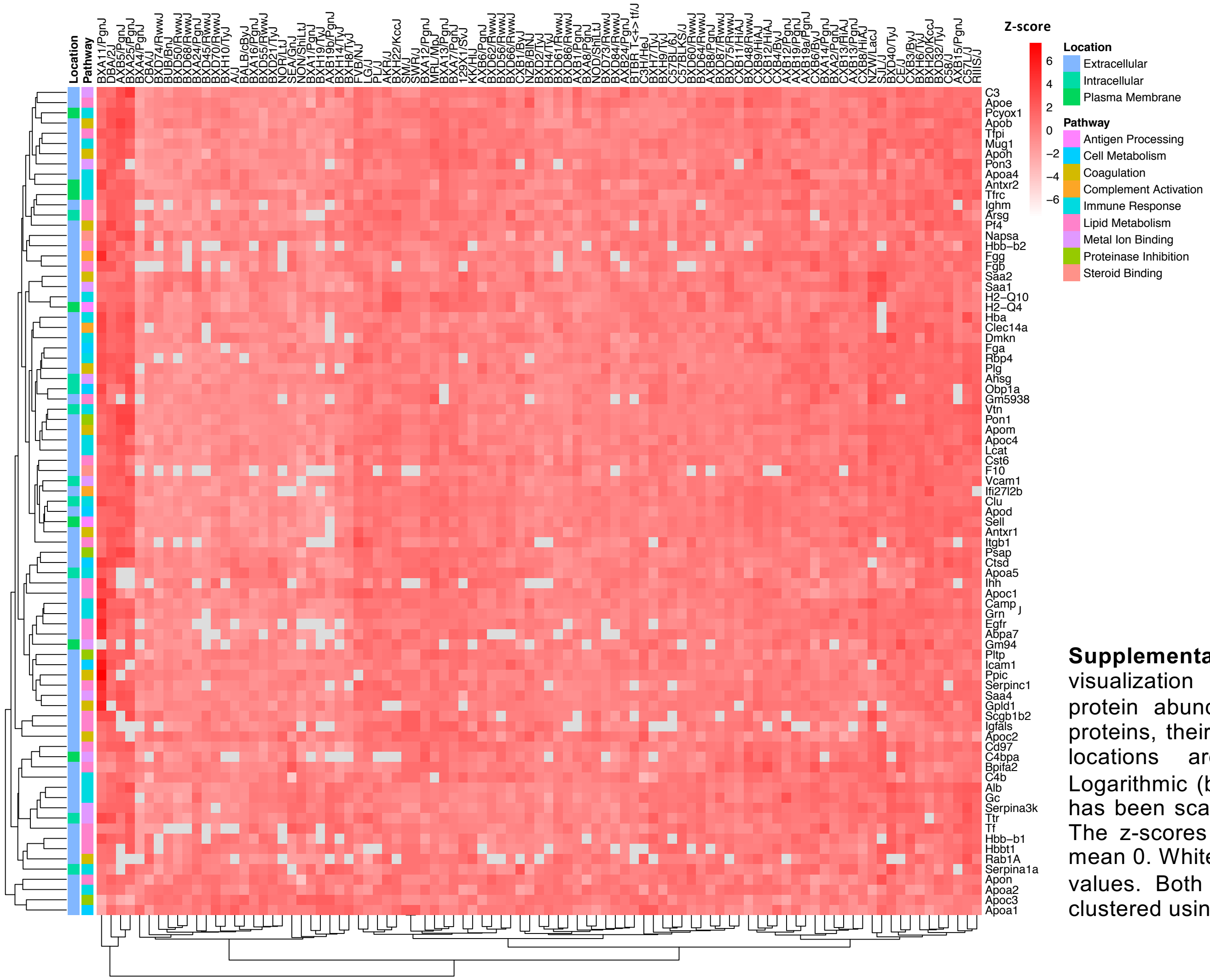

Supplemental Figure 8: The heatmap visualization of the yeast normalized HDL protein abundances across 93 strains. The proteins, their biological functions and cellular locations are represented. Following a Logarithmic (base 10) transformation the data has been scaled and centered across strains. The z-scores indicate the deviation from the mean 0. White squares represent not available values. Both the proteins and the strains were clustered using Euclidean distances.

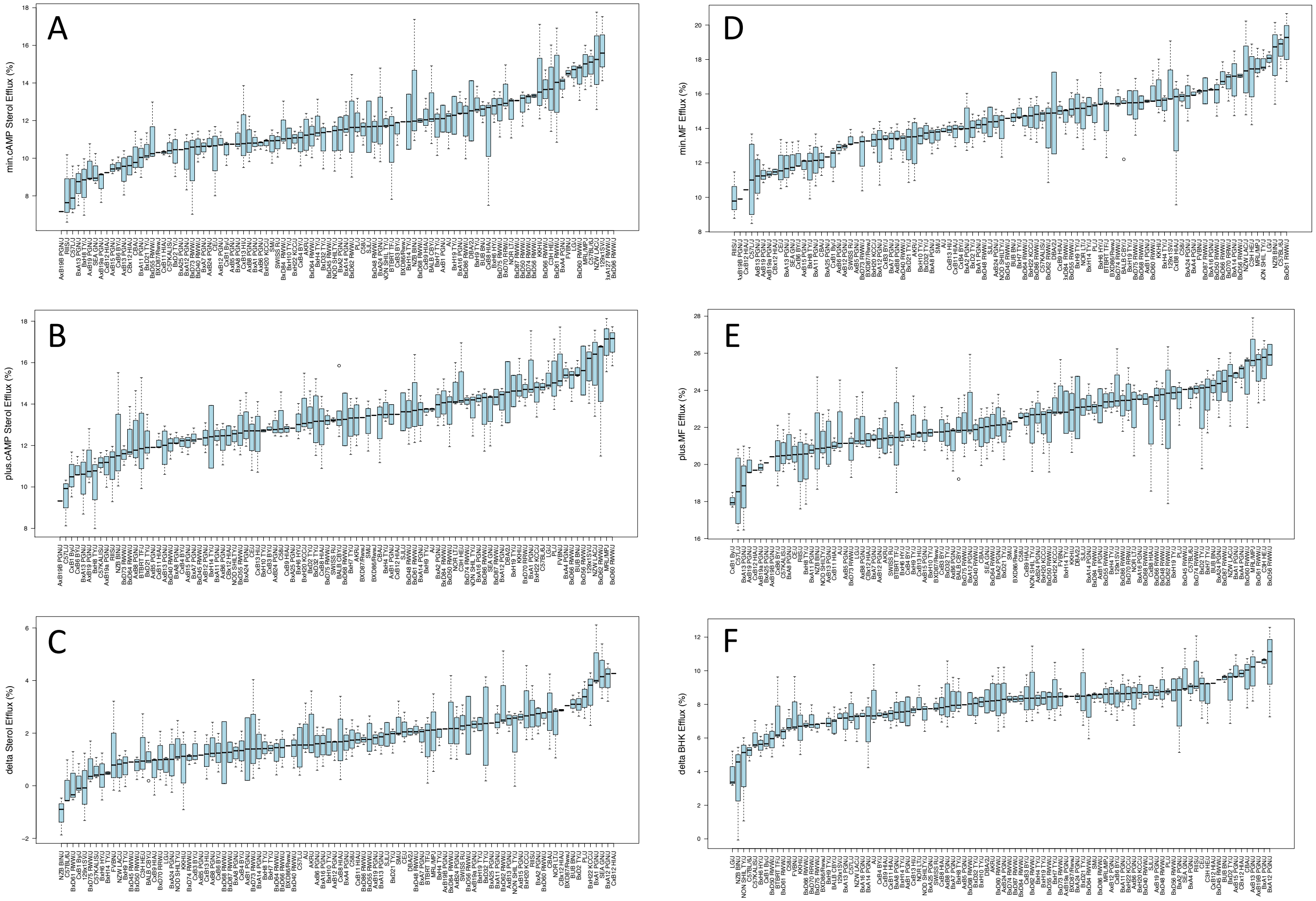

Supplemental Figure S6. Sterol Efflux distribution across strains. min.cAMP, plus.cAMP, delta.J774 are unstimulated, ABCA1 upregulated and ABCA1 specific sterol efflux from J774 cells respectively (-C). min.MF, plus.MF, delta.BHK are unstimulated, ABCA1 upregulated and ABCA1 specific sterol efflux from BHK cells respectively (D-F). All the measures are in duplicate from PEG depleted plasma collected from 1-5 mice for each strain.

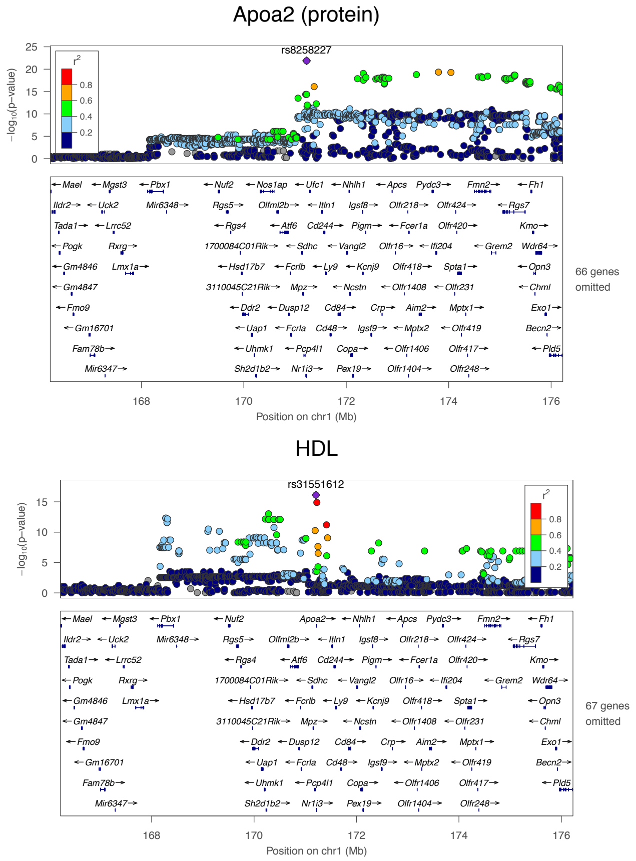

Supplemental Figure S7. Overlap of the loci regulating APOA2 and HDL-C.

Supplemental Table S2. Distal hepatic eQTL and HDL protein pQTL

		eQTL			pQTL		
**Gene symbol**	**gene location**	**rsID**	**location**	**pvalue**	**rsID**	**location**	**pvalue**
Acta2	chr19:34241091-34255336	rs33249546	chr12:107784606	3.19E-06	rs26854790	chr11:34507244	1.79E-05
Ahsg	chr16:22892042-22899438	-	-	-	rs27025295	chr11:102513585	6.96E-05
Alb	chr5:90460889-90476603	rs31797608	chr15:85144357	8.43E-06	rs32467664	chr4:149728061	8.13E-05
Antxr1	chr6:87133854-87335775	rs36880152	chr6:81882442	1.93E-06	rs50132559	chr19:33023700	3.38E-05
Antxr2	chr5:97884688-98030962	rs28126164	chr4:89835209	2.57E-06	rs33293355	chr15:44429881	1.49E-05
Apoa1	chr9:46228580-46230466	rs32772192	chr1:181446747	1.59E-06	rs36722533	chr5:114054566	1.76E-04
Apoa2	chr1:171225054-171226379	rs33710425	chr9:72868067	9.06E-07	rs3686444	chr1:176311409	4.58E-14
Apoa4	chr9:46240696-46243459	rs30020828	chr18:80220795	3.86E-07	rs33627742	chr7:136794307	6.13E-06
Apoa5	chr9:46268633-46271919	rs32391759	chr7:12925987	7.83E-06	rs27669302	chr2:178650304	1.35E-06
Apob	chr12:7977648-8016835	-	-	-	rs36252881	chr7:17157035	2.01E-05
Apoc1	chr7:19689484-19692658	rs29990911	chr9:46573895	7.61E-08	rs27318817	chr2:161825532	1.16E-04
Apoc2	chr7:19671584-19681423	rs28126164	chr4:89835209	3.01E-06	rs6152725	chr15:40403951	3.58E-08
Apoc3	chr9:46232933-46235636	rs33130644	chr17:77057385	1.19E-06	rs27103735	chr11:99579260	2.54E-05
Apoc4	chr7:19678094-19681416	rs30918547	chr1:135837872	1.38E-09	rs26948185	chr11:48065640	4.90E-05
Apod	chr16:31296192-31314808	-	-	-	rs27964211	chr2:75062346	3.69E-04
Apoe	chr7:19696109-19699166	rs30108818	chr3:150771887	1.09E-06	rs28224497	chr11:42181156	2.09E-04
Apoh	chr11:108343354-108414396	rs13481215	chr11:107133182	1.65E-15	rs30649661	chr14:85871809	2.77E-05
Apom	chr17:35128997-35132050	rs33090761	chr17:33672870	2.50E-10	rs27642220	chr2:181174626	2.01E-04
Apon	chr10:128254131-128255901	rs32101443	chr5:112119471	6.14E-06	rs29774020	chr18:58212937	2.38E-06
Arsg	chr11:109473374-109573330	rs36419130	chr19:27237192	1.91E-06	rs29057199	chrX:59442756	3.85E-04
B2m	chr2:122147686-122153083	rs27438827	chr2:120924297	1.69E-15	rs3686227	chr1:185787602	8.91E-04
Camp	chr9:109847375-109849456	rs26926153	chr11:8092518	2.70E-07	rs36661068	chr3:97125819	6.01E-05
Cd97	chr8:83723251-83741326	-	-	-	rs32535471	chr7:36356962	2.33E-06
Clec14a	chr12:58264720-58269258	rs36279328	chr15:10935337	3.06E-06	rs31569850	chr15:45550945	2.90E-04
Clu	chr14:65968483-65981548	rs46776915	chr1:76977058	3.43E-08	rs3148702	chr7:30320377	2.88E-04
Creb3l3	chr10:81084324-81098874	rs3090957	chr16:45620289	2.64E-07	rs30692446	chr6:121728413	3.95E-06
Cst6	chr19:5344705-5349574	-	-	-	rs27419289	chr2:92090332	3.54E-07
Ctsd	chr7:142371148-142388038	-	-	-	rs33582176	chr12:114598004	1.84E-04
Dmkn	chr7:30763756-30781066	-	-	-	rs3716768	chr15:42625082	2.21E-04
F2	chr2:91625329-91636407	rs45837563	chr8:92821223	6.36E-07	rs29807878	chr3:30317921	3.74E-04
Fetub	chr16:22918382-22939766	rs45734186	chr15:48796498	3.08E-07	rs38497950	chr9:45456361	7.32E-04
Fga	chr3:83026153-83033615	rs27373341	chr2:151322820	4.90E-06	rs49665653	chr10:15717736	1.08E-04
Fgb	chr3:83042303-83049790	rs27373341	chr2:151322820	9.32E-08	rs27013471	chr11:119119713	1.04E-04
Fgg	chr3:83007896-83015049	rs31600615	chr15:79335511	6.14E-07	rs36661068	chr3:97125819	5.73E-06
Gc	chr5:89417511-89457898	rs27037797	chr11:96844820	3.72E-06	rs31223648	chr1:153790065	3.56E-05
Gpld1	chr13:24943152-24990753	-	-	-	rs36661068	chr3:97125819	7.90E-05
Grn	chr11:102430315-102437048	-	-	-	rs27368358	chr2:92020480	3.63E-04

H2-Q10	chr17:35470089-35474563	rs13482968	chr17:37131683	8.59E-41	rs36858919	chr19:13828213	5.05E-07
Icam1	chr9:21015960-21028797	rs37594915	chr19:15778814	3.39E-06	rs36661068	chr3:97125819	3.17E-04
Igfals	chr17:24878770-24882008	rs47878742	chr17:37087269	5.02E-06	rs32144076	chr7:110386487	1.81E-06
Ihh	chr1:74945319-74951651	-	-	-	rs4184357	chr16:48902971	2.44E-04
Itgb1	chr8:128685654-128733200	rs51374406	chr12:64789105	3.63E-06	rs50104850	chr6:115450887	6.70E-05
Lcat	chr8:105939551-105943382	-	-	-	rs27677536	chr2:181265611	1.24E-05
Mug1	chr6:121838541-121889057	-	-	-	rs27979523	chr2:75221995	4.38E-05
Napsa	chr7:44572432-44586862	-	-	-	rs32164737	chr7:29552540	7.48E-06
Obp1a	chrX:78085505-78091374	-	-	-	rs28277205	chr2:174386694	2.91E-04
Pcyox1	chr6:86386006-86397150	-	-	-	rs27529991	chr4:125355058	4.68E-05
Pf4	chr5:90772435-90773381	-	-	-	rs27642374	chr2:181116314	5.39E-05
Plg	chr17:12378609-12419384	rs27619641	chr4:135387675	1.75E-06	rs33468024	chr12:113826839	7.47E-06
Pltp	chr2:164839518-164857711	rs38870920	chr9:49969825	2.17E-06	rs27971647	chr2:71119784	1.38E-04
Pon1	chr6:5168090-5193946	rs31124585	chr3:42161472	6.42E-06	rs27499799	chr4:120761555	1.61E-05
Pon3	chr6:5220852-5256286	-	-	-	rs29829269	chr6:12796975	5.41E-06
Ppic	chr18:53406341-53418007	rs29850615	chr18:7766071	1.59E-08	rs36661068	chr3:97125819	2.90E-05
Psap	chr10:60277627-60302594	rs29170614	chr12:100443804	3.15E-06	rs27516360	chr4:120659954	2.75E-05
Rab10	chr12:3247430-3309969	rs31977226	chr7:24356524	8.81E-07	rs33497497	chr5:25445855	1.51E-04
Rbp4	chr19:38116620-38125321	rs29232569	chr13:95538693	7.75E-07	rs47135895	chr12:117094100	1.88E-04
Saa1	chr7:46740501-46742980	-	-	-	rs36355916	chr19:16510334	1.68E-07
Saa2	chr7:46751833-46754314	rs45654115	chr14:121548049	1.23E-06	rs36608891	chr18:64690680	5.44E-12
Saa4	chr7:46728017-46732543	rs29737430	chr9:41680673	5.68E-09	rs27665189	chr2:180274844	3.00E-05
Sell	chr1:164062076-164080785	-	-	-	rs27964211	chr2:75062346	8.22E-05
Serpina3k	chr12:104338486-104345741	rs27900188	chr2:48696367	7.36E-08	rs37260091	chr10:14030671	2.44E-05
Serpinc1	chr1:160978606-161003010	rs50125449	chr14:47256172	8.73E-06	rs28042579	chr2:59173843	5.34E-04
Tfpi	chr2:84432855-84476775	rs27398927	chr2:89930200	6.84E-06	rs32170249	chr7:46464534	1.36E-04
Tfrc	chr16:32608920-32632794	rs37655376	chr18:70771803	5.53E-07	rs29339441	chr10:120721000	3.00E-06
Ttr	chr18:20665250-20674324	rs31496994	chr7:136390392	7.02E-07	rs29051466	chrX:67329987	8.81E-04
Vcam1	chr3:116110020-116129688	-	-	-	rs29055848	chrX:60937611	5.82E-05
Vtn	chr11:78499091-78502324	rs32054038	chr7:47248355	1.47E-07	rs27642377	chr2:181116206	5.65E-05

Supplemental Table S3. Local adipose eQTL and HDL apoprotein pQTL

		local eQTL			local pQTL		
**Gene symbol**	**Gene location**	**rsID**	**location**	**p-value**	**rsID**	**location**	**p-value**
Apoa2	chr1:171225054-171226379	-	-	-	rs8258227	chr1:171225758	1.63E-22
Apoc1	chr7:19689484-19692658	rs32424040	chr7:19760563	4.98E-04	-	-	-
Apoc2	chr7:19671584-19681423	rs32424040	chr7:19760563	9.75E-05	rs32420618	chr7:19571593	7.92E-05
Apoc3	chr9:46232933-46235636	rs30024591	chr9:45614034	1.77E-07	rs48945377	chr9:46673334	5.41E-04
Cd97	chr8:83723251-83741326	rs6244441	chr8:83006485	1.63E-05	-	-	-
Cst6	chr19:5344705-5349574	rs37117750	chr19:5255634	4.61E-04	-	-	-
H2-Q10	chr17:35470089-35474563	rs8239672	chr17:34659014	6.21E-11	rs33061052	chr17:35078160	2.99E-07
Saa1	chr7:46740501-46742980	-	-	-	rs3154655	chr7:46985523	1.19E-05
Saa2	chr7:46751833-46754314	-	-	-	rs3154655	chr7:46985523	1.09E-10
Ttr	chr18:20665250-20674324	rs52621808	chr18:19774644	4.10E-04	-	-	-
Vcam1	chr3:116110020-116129688	rs30451190	chr3:117000199	7.10E-04	-	-	-

Supplemental Table S4. Potential causal interactions based on local pQTL analyses

Gene symbol	gene location	rsID	SNP location	p-value	effect size
Apoc2	chr7:19671584-19681423	rs32420618	chr7:19571593	2.19E-04	0.0132712
Saa2	chr7:46751833-46754314	rs32420618	chr7:19571593	1.96E-03	0.0400445
H2-Q10	chr17:35470089-35474563	rs33061052	chr17:35078160	9.58E-07	-0.0874375
Apob	chr12:7977648-8016835	rs33061052	chr17:35078160	1.74E-02	0.131756
Apoe	chr7:19696109-19699166	rs33061052	chr17:35078160	3.00E-02	0.0868838
Apoc3	chr9:46232933-46235636	rs48945377	chr9:46673334	5.56E-04	-0.0625709
Podxl	chr6:31519488-31563981	rs48945377	chr9:46673334	4.09E-03	0.0145041
Fetub	chr16:22918382-22939766	rs48945377	chr9:46673334	5.47E-03	0.0121822
Apoc2	chr7:19671584-19681423	rs48945377	chr9:46673334	5.87E-03	-0.00916968
Itgb3	chr11:104608000-104670476	rs48945377	chr9:46673334	1.74E-02	0.00912179
Tor3a	chr1:156653617-156674356	rs48945377	chr9:46673334	2.64E-02	0.00647641
Antxr2	chr5:97884688-98030962	rs48945377	chr9:46673334	2.92E-02	0.0113798
Tmsb4x	chrX:167207093-167209315	rs48945377	chr9:46673334	3.45E-02	0.00992826
Plg	chr17:12378609-12419384	rs48945377	chr9:46673334	4.13E-02	0.0346354
Gc	chr5:89417511-89457898	rs48945377	chr9:46673334	4.86E-02	0.0194774
Apoa2	chr1:171225054-171226379	rs8258227	chr1:171225758	1.32E-22	-0.41335
Apoc3	chr9:46232933-46235636	rs8258227	chr1:171225758	7.15E-03	0.0520746
Itgb3	chr11:104608000-104670476	rs8258227	chr1:171225758	1.96E-02	-0.00944052

The Supplementary Tables listed below are submitted as data files.

Supplementary Table S1. Summary of HDL proteins per strain.

Supplementary Table S5. The correlations for the HDL metrics.

Supplemental Table S6. Association of HDL Proteins with Clinical Traits in HMDP.
